# Toe walking: A regional analysis of TTN variants between c.10,000 and c.99,999 in a cohort of children

**DOI:** 10.1016/j.gmg.2026.100123

**Published:** 2026-08-01

**Authors:** David Pomarino, Amel Sidi Athmane, Bastian Fregien, Alexander Nazarkin, Kevin M. Rostásy

**Affiliations:** a Pomarino Praxis für Ganganomalien, Hamburg, Germany; b ORTHOMAX, orthopädische üBAG GbR Langenhagen, Germany; c Fakultät für Gesundheit (Department für Humanmedizin), Universität Witten/Herdecke, Witten/Herdecke, Germany

**Keywords:** Genetic Testing, Toe Walking, Neurology, Pediatrics, TTN

## Abstract

**Background:**

Persistent toe walking is traditionally considered idiopathic when no neurological or orthopedic cause is identified. Emerging evidence suggests genetic contributors, including TTN, but genotype–phenotype relationships remain poorly understood in affected children.

**Methods:**

This retrospective descriptive study evaluated children with persistent toe walking assessed between November 2022 and April 2026 at a specialized physiotherapy practice. All participants underwent standardized clinical assessment and targeted next-generation sequencing. Individuals carrying TTN variants within the c.10,000–99,999 region were stratified into nine predefined cDNA intervals. Demographic characteristics, gait features, musculoskeletal findings, neurological signs, and variant distributions were summarized descriptively.

**Results:**

The cohort comprised 581 individuals carrying 509 TTN variants. Most variants were classified as variants of uncertain significance, while likely pathogenic variants were uncommon. Male predominance was observed across all intervals. Mean age at assessment, age at independent walking, age at toe-walking onset, and toe-walking frequency were highly consistent between groups. Reduced ankle dorsiflexion was the most frequent examination finding, affecting 88.2–97.4% of participants. Muscle pain, cramps, fatigue, spinal abnormalities, heel-walking impairment, pes cavus, tremor, and other associated features demonstrated modest variation across intervals without a consistent regional pattern. Several recurrent TTN variants were identified, most notably c.32624 C>T, c.48727 C>T, and c.73825 G>C.

**Conclusions:**

Children carrying TTN variants within the c.10,000–99,999 region exhibited broadly similar clinical phenotypes characterized by persistent toe walking and reduced ankle dorsiflexion. Stratification by cDNA location did not reveal clear genotype–phenotype clustering, suggesting limited predictive value of variant position alone and supporting the need for functional and segregation-based studies in future research.

## Introduction

Persistent toe walking is a common gait abnormality in children and has traditionally been classified as idiopathic when no underlying neurological or orthopedic cause can be identified. However, growing evidence suggests that genetic factors may contribute to its development in at least a subset of affected individuals [1], [2], [3].

Among the genes increasingly identified in children with toe walking and related gait abnormalities is TTN. Variants in TTN have been associated with a broad spectrum of skeletal muscle and cardiac disorders, although their clinical interpretation remains challenging [4]. Establishing genotype–phenotype correlations is particularly difficult because TTN harbors extensive genetic variation within the general population, and the clinical significance of individual variants may be influenced by factors such as transcript expression, exon usage, protein domain location, and inheritance pattern [5].

TTN encodes titin, the largest known human protein, which spans the sarcomere from the Z-disc to the M-line and contributes to muscle elasticity, structural integrity, and intracellular signaling [5], [6]. Owing to its large size and complex genomic organisation, TTN contains numerous rare variants, many of which are classified as variants of uncertain significance.

Given the large size of the gene, evaluating TTN variants as a single group may obscure potential differences in variant distribution and associated clinical findings. Although TTN has been extensively investigated in cardiomyopathies and neuromuscular disorders, limited information is available regarding the distribution of TTN variants in children presenting primarily with persistent toe walking. Furthermore, whether variants located in different regions of the gene are associated with distinct clinical characteristics remains largely unexplored.

The present study is part of a larger investigation of TTN variant distribution across the gene. In this study, variants located within the c.10,000–c.99,999 region were grouped into predefined cDNA intervals to facilitate a structured descriptive analysis. The aim was to characterize the distribution of TTN variants within this region and to explore potential differences in clinical features across variant groups. This study is descriptive in nature and is not intended to establish causality, but rather to identify patterns that may inform future genotype–phenotype investigations.

## Methods

### Study design and population

This retrospective descriptive study evaluated children presenting with persistent toe walking at a physiotherapy practice specializing in the assessment and management of toe walking. The study cohort was derived from approximately 2000 patients who underwent a standardized clinical assessment protocol.

Patients assessed between November 2022 and April 2026 were screened for inclusion. Clinical and genetic data available at the time of database closure were reviewed for all eligible participants.

Patients were referred through established clinical pathways, either for initial evaluation or following assessment at other specialist centers. All participants underwent a comprehensive diagnostic work-up designed to identify potential neurological, neuromuscular, orthopedic, or developmental causes of toe walking.

### Eligibility criteria

Inclusion was restricted to children with persistent toe walking in whom no specific neurological, neuromuscular, or orthopedic etiology was identified following structured clinical evaluation. Patients were referred through established clinical pathways, either directly for primary evaluation at our centre or following prior assessment at specialised centres. Inclusion required the absence of a defined neurological, neuromuscular, or orthopedic cause of toe walking after comprehensive evaluation. As part of routine clinical assessment, all included patients underwent genetic testing independent of any prior knowledge of gene status.

Patients with an established alternative diagnosis considered capable of explaining the gait abnormality were excluded. Examples included cerebral palsy, autism spectrum disorder, muscular dystrophy, tethered cord syndrome, and structural deformities not attributable to neuropathic mechanisms.

### Genetic analysis and variant selection

For the present analysis, patients carrying TTN variants were identified from the study database. Variants were grouped according to predefined cDNA intervals to facilitate descriptive evaluation of their distribution and associated clinical characteristics. The current study focuses on TTN variants located within the c.10,000–99,999 region.

### Ethics

The study was conducted in accordance with the principles of the Declaration of Helsinki and complied with all applicable institutional and legal requirements. Ethical approval was granted by the Ethics Committee of the Deutschen Verbandes für Physiotherapie at the Physio-Akademie, Wremen, Germany (Project No. 2025–02).

**Fig. 1:**
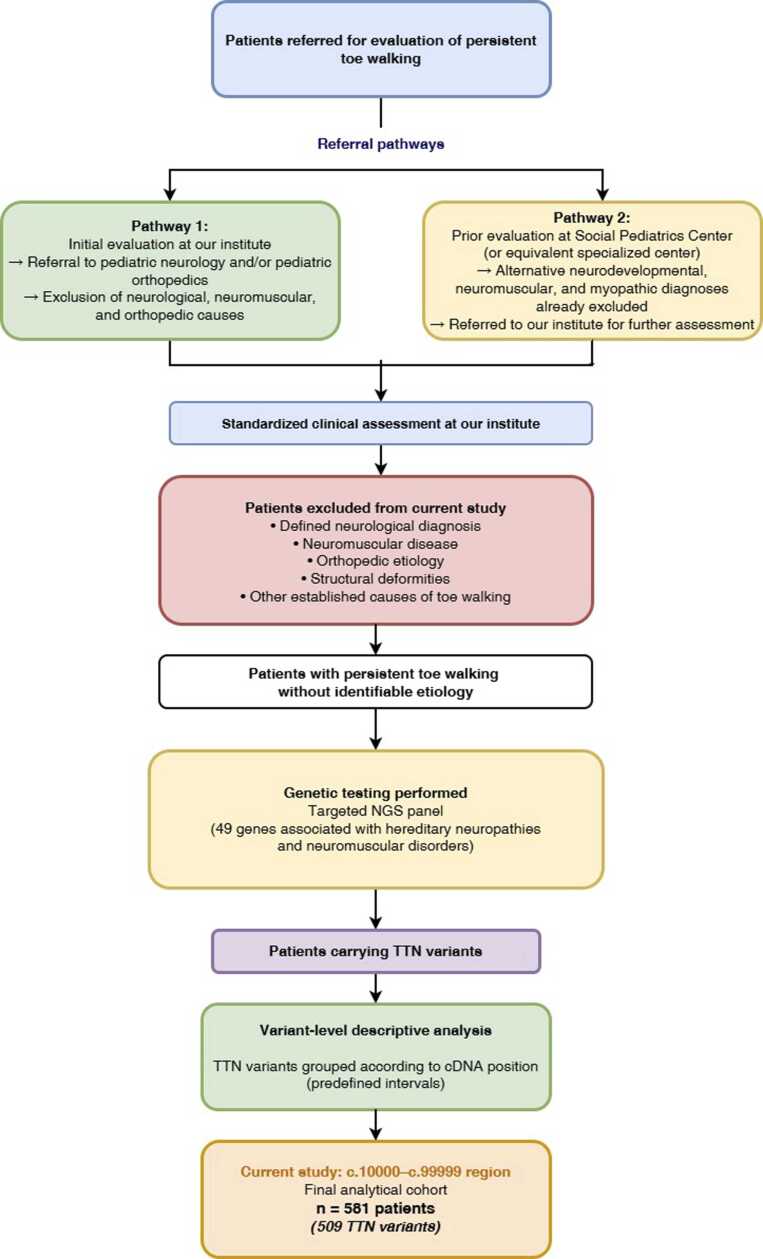
Flowchart of patient referral, assessment, genetic testing, and selection of the final study cohort.

A schematic overview of patient referral, clinical assessment, eligibility screening, genetic testing, and cohort selection is shown in Fig. 1.

### Clinical examination and assessment procedures

All participants underwent a standardized clinical evaluation at our physiotherapy practice. Examinations were performed independently of genetic testing results, and assessors remained unaware of variant status throughout the clinical assessment process. A predefined protocol was used for all patients and included collection of medical history, postural and musculoskeletal examination, gait assessment, and neurological evaluation.

### Medical history

A structured history was obtained from parents or primary caregivers. Information collected included demographic characteristics (age and sex), age at independent walking, age at onset of toe walking, and the pattern and progression of the gait abnormality. Family history of toe walking was documented when present.

Additional information included muscular symptoms such as pain, cramps, or weakness, developmental features including speech or language delay, social or behavioral abnormalities, and the presence of visual impairment.

### Musculoskeletal and postural assessment

A clinical inspection was performed to identify structural and postural abnormalities. Features assessed included lumbar hyperlordosis, pes cavus, foot drop, pectus excavatum, pectus carinatum, clinodactyly, and brachydactyly.

### Balance and functional motor assessment

Balance was evaluated using a single-leg stance task. Failure to maintain the position independently for at least five seconds was considered abnormal.

Functional motor performance was additionally assessed using heel walking. Inability to sustain heel walking was interpreted as evidence of impaired ankle dorsiflexor function.

### Ankle range of motion

Ankle dorsiflexion and plantarflexion were evaluated as part of the routine clinical examination. Assessments were performed with patients in the supine position and the knees extended to facilitate differentiation between gastrocnemius and soleus muscle contributions. Range of motion findings were categorized as normal or reduced based on visual inspection.

### Neurological examination

Neurological assessment included evaluation of patellar deep tendon reflexes and screening for tremor. Patellar reflexes were elicited with the patient seated and the lower limbs relaxed. The patellar tendon was tapped using a reflex hammer, and responses were categorized as normal, reduced, or absent. Tremor assessment was performed with the arms extended anteriorly and the palms facing downward. Patients were instructed to repeatedly open and close their hands. Tremor was recorded when rhythmic oscillatory movements were observed during or immediately following the maneuver.

### Genetic analysis

All participants included in the study underwent genetic testing using a standardized targeted next-generation sequencing (NGS) protocol. To maintain methodological consistency, patients analyzed using alternative approaches, including whole-exome sequencing, were excluded.

Genetic analysis was performed using a validated 49-gene panel (Thermo Fisher Scientific) targeting genes associated with hereditary neuropathies, myopathies, and related neuromuscular disorders that may present with gait abnormalities (Supplementary Table 1). Gene selection was guided by Human Phenotype Ontology (HPO) terms relevant to neuropathic and myopathic phenotypes.

Saliva samples were collected during routine clinical evaluation. DNA extraction was performed using standard laboratory procedures on an automated Promega Maxwell CSC system with the CSC Blood Kit or with the QIAamp DNA Blood Mini Kit. Sequencing was conducted using an amplicon-based custom panel on the Ion Torrent platform (Thermo Fisher Scientific, Waltham, MA, USA). Target regions included all coding exons and exon–intron boundaries, with intronic regions evaluated up to ±10 base pairs from the exon border.

Sequence data were analyzed using the SeqNext module of SeqPilot software (version 5.4.3). Variants were considered for interpretation only when sequencing coverage was at least 30 × and the variant allele read frequency exceeded 20%.

Variant classification followed a five-tier system comprising benign (Class 1), likely benign (Class 2), variant of uncertain significance (Class 3), likely pathogenic (Class 4), and pathogenic (Class 5) categories according to ACMG/AMP recommendations [7]. Variants classified as benign or likely benign were filtered during bioinformatic processing and were not included in the final dataset. Only pathogenic, likely pathogenic, and variants of uncertain significance (VUS) were retained for analysis.

Variant interpretation was supported, where necessary, by in silico prediction tools including MutationTaster, PolyPhen−2, and Mutation Assessor. Additional evidence was obtained from publicly available and disease-specific databases, including HGMD Professional, LOVD-IARC, dbSNP, ClinVar, and locus-specific databases when applicable.

Copy-number variation (CNV) analysis was incorporated into the diagnostic workflow. Multiplex Ligation-dependent Probe Amplification (MLPA) was performed using validated probe sets targeting PMP22, ATL1, SPAST, REEP1, SPG7, and SPG11 to confirm suspected deletions or duplications identified during sequencing and to detect clinically relevant CNVs.

Testing was performed by the accredited diagnostic laboratory Labor Dr. Heidrich & Colleagues (Hamburg, Germany). According to laboratory validation data, the analytical sensitivity for clinically relevant variants exceeded 96%. Segregation analysis was not routinely performed because of practical and financial considerations.

The applied sequencing strategy was limited to targeted coding regions and adjacent intronic sequences. Consequently, variants located in untranslated regions, regulatory elements, regions with high sequence homology, repeat regions, or other non-targeted genomic areas may not have been detected. In addition, single-exon or whole-gene copy-number changes outside the validated assay scope and low-level mosaic variants cannot be completely excluded. As scientific knowledge evolves, variant classifications may also change over time based on emerging evidence.

### Variant grouping strategy

Given the size and complexity of the TTN gene, variants were analyzed according to their cDNA location. Predefined cDNA intervals were created to facilitate descriptive comparison of variant distribution and associated clinical characteristics across different regions of the gene.

Variants were treated as independent observations. Consequently, Individuals carrying one or more TTN variants classified as pathogenic, likely pathogenic or variants of uncertain significance were included. Benign and likely benign variants were excluded during laboratory filtering. When multiple TTN variants were present within the same individual, each variant was assigned to its respective cDNA interval.

### Statistical analysis

Statistical analyses were descriptive in nature and were performed using LibreOffice Calc (version 25.2.5.2).

Categorical variables are presented as frequencies and percentages. Percentages were calculated using available-case denominators, with missing observations excluded from individual analyses. Continuous variables are summarized using means and standard deviations where applicable. Exact 95% confidence intervals for proportions were calculated using Fisher's exact method when appropriate. Because the objective of the study was exploratory and descriptive, no formal hypothesis testing, multivariable analyses, correction for multiple comparisons, or inferential genotype–phenotype analyses were performed. The study flowchart was generated using diagrams.net (draw.io), version 26.2.2.

## Results

### Cohort overview

The study cohort comprised 581 individuals carrying 509 distinct TTN variants. Of these, 381 (65.6%) were male and 200 (34.4%) were female. The mean age at assessment was 7.3 years.

### Cohort distribution

A total of 581 individuals carrying 509 TTN variants within the c.10,000–c.99,999 region were included and stratified into nine predefined cDNA intervals. The largest subgroup was c.10,000–19,999 (n = 127), whereas the smallest was c.20,000–29,999 (n = 76). Sample sizes in the remaining groups ranged from 77 to 118 individuals.

Across all intervals, most variants were classified as variants of uncertain significance (VUS), accounting for 95.8–100% of variants. Likely pathogenic (LP) variants were uncommon and represented between 0% and 4.2% of variants within individual intervals.

Male predominance was observed in all groups, with males comprising 60.2–71.1% of participants.

A comprehensive summary of demographic characteristics, clinical findings, and variant distribution across all cDNA intervals is provided in Supplementary Table 2. The complete anonymized genetic dataset, including all TTN variants identified in the study cohort, is presented in Supplementary Table 3.

### Distribution of recurrent TTN variants

**Fig. 2:**
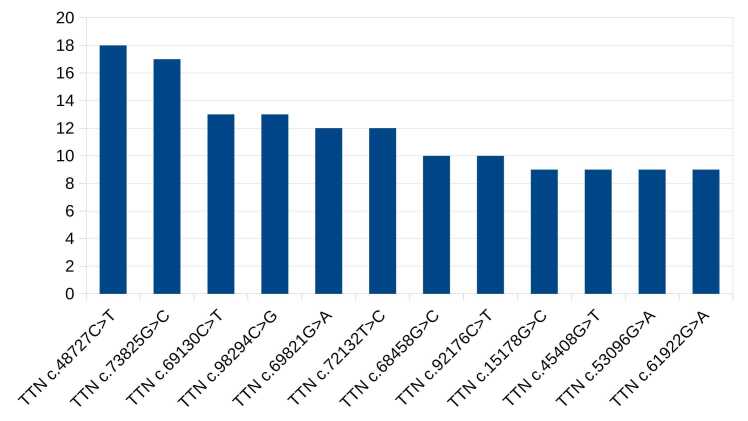
Distribution of the most frequently identified TTN variants in the study cohort.

Several recurrent TTN variants were identified within the study cohort. The most frequently observed variants were TTN c.32624 C>T and TTN c.48727 C>T (n = 18 each), followed by TTN c.73825 G>C (n = 17). Other recurrent variants were observed at lower frequencies, ranging from 9 to 13 occurrences. The distribution of the most frequently identified TTN variants is presented in Fig. 2.

### Age at presentation and toe-walking characteristics

The mean age at assessment was relatively consistent across groups, ranging from 6.5 to 7.9 years.

Age at independent walking showed little variation between intervals, with mean values ranging from 13.2 to 13.9 months. Similarly, the mean age at onset of toe walking remained stable across groups (24.0–28.8 months).

The proportion of individuals whose toe walking began at approximately the same time as independent walking ranged from 57.5% to 69.3%. The highest proportion was observed in the c.70,000–79,999 interval (69.3%).

Toe-walking frequency was remarkably consistent across groups, affecting more than 80% of the day in all intervals (81.4–85.3%).

When progression of toe walking was evaluated, most participants reported either stable symptoms or worsening over time. Improvement was reported less frequently, ranging from 7.6% to 15.8%.

### Pain and functional symptoms

Calves and feet represented the most common locations of pain across all intervals. Reported muscle pain ranged from 15.5% to 30.3%, with the highest prevalence observed in the c.20,000–29,999 interval.

Muscle cramps were reported by 15.7–30.3% of participants, while fatigue was reported by 13.4–26.3%. Both symptoms were most prevalent in the c.20,000–29,999 interval.

Speech or language delay was reported in approximately half of the cohort, ranging from 37.7% to 50.4%. Social or behavioral abnormalities were less common, affecting 8.8–15.5% of participants. Visual impairment was reported in 5.9–13.2% of participants.

### Neuromuscular examination findings

Spinal abnormalities, primarily lumbar hyperlordosis, were observed in 13.0–30.0% of participants. The highest prevalence was recorded in the c.60,000–69,999 interval (30.0%) and the lowest in the c.80,000–89,999 interval (13.0%). Single cases of scoliosis were identified in selected groups and are indicated by an asterisk in Supplementary Table 2.

Balance impairment was present in 9.0–19.0% of individuals and showed no clear regional clustering.

Heel walking impairment varied substantially between groups, ranging from 0% in the c.10,000–19,999 interval to 14.3% in the c.80,000–89,999 interval.

Reduced ankle dorsiflexion was the most consistent examination finding and was present in 88.2–97.4% of participants across all intervals.

Pes cavus was reported in 8.5–17.8% of participants, with the highest frequency observed in the c.70,000–79,999 interval.

Deep tendon reflex abnormalities were infrequent and demonstrated no obvious regional pattern. Both reduced and increased reflexes were observed at low frequencies across all intervals.

### Musculoskeletal features

Brachydactyly/clinodactyly were the most prevalent associated musculoskeletal findings, occurring in 63.2–72.4% of individuals in most intervals.

Pectus excavatum was present in 18.2–28.1% of participants, while pectus carinatum occurred in 11.7–22.8%.

Tremor was reported in 2.4–18.6% of individuals, with the highest prevalence observed in the c.60,000–69,999 interval.

Hyperhidrosis and clonus were uncommon findings and generally occurred in fewer than 5% of participants. Trendelenburg gait was reported in approximately 5–8% of individuals across most intervals.

## Discussion

This study provides a descriptive analysis of TTN variants located within the c.10,000–99,999 region in a large cohort of children presenting with persistent toe walking. By stratifying variants into predefined cDNA intervals, we sought to determine whether regional clustering within TTN was associated with recognizable clinical differences. Overall, the findings demonstrated substantial phenotypic overlap across all intervals, with no clear evidence of region-specific clinical profiles.

We intentionally grouped variants according to cDNA position rather than protein domain because the objective of the present study was to provide an initial descriptive overview of regional variant distribution. Future studies incorporating functional protein domains and transcript-specific expression may provide more biologically relevant genotype–phenotype analyses.

Several core features were consistently observed throughout the cohort, including early onset toe walking, high toe-walking frequency, reduced ankle dorsiflexion, and a predominance of male patients. Age at independent walking and age at toe-walking onset were remarkably similar across groups, suggesting that variants located within different regions of the gene are associated with broadly comparable clinical presentations in this cohort.

Although modest variation was observed for certain clinical characteristics, including muscle symptoms, spinal abnormalities, heel-walking impairment, and selected musculoskeletal findings, these differences did not demonstrate a consistent pattern across cDNA intervals. Furthermore, no interval was characterized by a distinct phenotype that clearly differentiated it from neighbouring regions. Taken together, these observations suggest that variant location within the c.10,000–99,999 region alone is insufficient to explain clinical variability.

One notable finding was the identification of several recurrent TTN variants that appeared repeatedly throughout the cohort. Whether these recurrent variants represent population-specific findings, frequently encountered benign background variation, or variants with potential clinical relevance cannot be determined from the present data. Nevertheless, their repeated occurrence highlights the importance of careful variant interpretation when evaluating TTN findings in clinical practice.

The absence of a clear genotype–phenotype relationship is not unexpected given the biological complexity of TTN. As the largest protein-coding gene in the human genome, TTN exhibits substantial genetic variation within the general population, and the clinical significance of individual variants may depend on multiple factors including transcript expression, exon usage, protein domain localization, inheritance pattern, and variant type. Consequently, categorization based solely on cDNA position may not adequately capture the functional consequences of individual variants.

Interpretation is further complicated by the genetic architecture of the study cohort. Some individuals carried multiple TTN variants located in different regions of the gene, while others also harbored variants in additional genes included in the neuromuscular testing panel. As a result, the observed phenotype cannot necessarily be attributed to a single TTN variant or genomic region. Potential cumulative, modifying, or interacting effects between variants could not be assessed within the scope of the present analysis.

A major strength of this study is the relatively large cohort size and the use of standardized clinical and genetic assessment protocols. Clinical evaluations were performed independently of genetic testing results, reducing the likelihood of ascertainment bias during phenotype documentation. In addition, the region-based analytical framework allowed systematic assessment of variant distribution across a substantial proportion of the TTN coding sequence.

Several limitations should be acknowledged. First, the study was descriptive and exploratory in nature and was not designed to establish causality. Second, most identified variants were classified as variants of uncertain significance, and future reclassification may alter interpretation of the findings. Third, the absence of a control population prevents assessment of whether the observed TTN variants occur more frequently than expected in the general population. Fourth, segregation analysis was not routinely available, limiting interpretation of inheritance patterns and preventing assessment of phase relationships among multiple variants. Fifth, some participants carried variants in genes other than TTN, which may have contributed to the clinical presentation. Finally, electrophysiological investigations were not routinely performed and therefore subtle neuropathic involvement cannot be excluded.

In conclusion, children carrying TTN variants within the c.10,000–99,999 region demonstrated a largely consistent clinical phenotype characterized by persistent toe walking and reduced ankle dorsiflexion. Regional grouping of variants did not reveal clear genotype–phenotype clustering, suggesting that cDNA location alone has limited value for predicting clinical presentation within this portion of the gene. Further studies incorporating segregation analysis, functional characterization, and comprehensive genomic approaches will be required to clarify the contribution of TTN variation to toe walking and related gait abnormalities.

## Funding Statement

This research did not receive any specific grant from any funding agency in the public, commercial, or not-for-profit sectors.

## Ethical statement

This study was conducted in accordance with the ethical principles of the World Medical Association Declaration of Helsinki. All procedures were performed in compliance with relevant laws and institutional guidelines and were approved by the ethical board of the.

Deutschen Verbandes für Physiotherapie an der Physio-Akademie in Wremen, Germany (project number 2025–02).

Prior to participation, written informed consent was obtained from all subjects. The consent process included explanations of the study's purpose, procedures, and any potential implications of the results. All data collected were formally anonymized to protect participant confidentiality.

This manuscript was prepared in accordance with the International Committee of Medical Journal Editors (ICMJE) recommendations.

## CRediT authorship contribution statement

David Pomarino contributed to the conceptualization of the study, data curation, and formal analysis. Amel Sidi Athmane contributed to data curation and investigation and drafted the original manuscript. Bastian Fregien and Alexander Nazarkin contributed to the study methodology and validation. Kevin M. Rostásy contributed to formal analysis, supervision, validation, and manuscript review and editing. All authors reviewed and approved the final manuscript.

## Declaration of Generative AI and AI-assisted technologies in the writing process

Generative AI Deepseek was used in the writing phase of this manuscript exclusively for linguistic polishing and enhancing clarity. All scientific reasoning, data analysis, and intellectual substance remain the sole contribution of the authors. The AI was not employed in the research process itself.

## Conflict of Interest

The Authors declare that there is no conflict of interest.

## Data Availability

Raw next-generation sequencing data were generated as part of routine clinical diagnostics and are not publicly available because of ethical, privacy, and institutional restrictions governing human genetic data. De-identified variant-level data supporting the findings of this study are provided in Supplementary Table 3. Additional anonymized data may be made available from the corresponding author upon reasonable request and subject to ethical approval.

## Footnotes

Appendix A Supplementary data associated with this article can be found in the online version at doi:10.1016/j.gmg.2026.100123.
