# Supplementary material for: Toe walking: A regional analysis of TTN variants between c.10,000 and c.99,999 in a cohort of children
Source: Glob Med Genet. 2026 Aug 1;13(3):100123. doi: 10.1016/j.gmg.2026.100123 (PMC13471197; doi:10.1016/j.gmg.2026.100123)
Supplement: Supplementary file 1 [file mmc1.docx]

Table 1: List of the 49 genes analyzed in the hereditary neuropathy NGS panel (adapted from Pomarino et al., 2023, PMCID: PMC10121371^[1]^)

| Gene | Transcript (RefSeq/LRG*) |
| --- | --- |
| AIFM1 | NM_004208.3 |
| ALS2 | NM_020919.3 |
| ATM | LRG_135t1 |
| ATXN1 | LRG_863t1 |
| ATXN2 | NM_002973.3 |
| ATXN3 | NM_004993.5 |
| ATXN7 | NM_000333.3 |
| CACNA1A | LRG_7t1 |
| CAV3 | LRG_329t1 |
| CHRNE | LRG_1254t1 |
| CLCN1 | NM_000083.2 |
| COL6A2 | LRG_476t1 |
| COL6A3 | LRG_473t1 |
| CREBBP | NM_004380.3 |
| DHTKD1 | NM_018706.6 |
| EGR2 | LRG_234t1 |
| EPHB4 | NM_004444.4 |
| FBLN5 | LRG_240t1 |
| FGD4 | NM_139241.3 |
| FXN | NM_000144.4 |
| GARS1 | NM_002047.3 |
| GDAP1 | LRG_244t1 |
| IQSEC2 | NM_001111125.2 |
| KCNC3 | NM_004977.2 |
| KMT2C | NM_170606.2 |
| LITAF | LRG_253t1 |
| MED25 | LRG_368t1 |
| MFN2 | LRG_255t1 |
| MORC2 | NM_001303256.2 |
| MPZ | LRG_256t1 |
| NAGLU | NM_000263.3 |
| NDRG1 | LRG_258t1 |
| NEFL | LRG_259t1 |
| OPA1 | LRG_337t2 |
| PMP22 | LRG_263t1 |
| POLG | LRG_765t1 |
| PRX | LRG_265t2 |
| PYGM | NM_005609.3 |
| RETREG1 | LRG_363t1 |
| SATB2 | NM_015265.3 |
| SBF1 | NM_002972.3 |
| SBF2 | LRG_267t1 |
| SH3TC2 | LRG_371t1 |
| **SPTLC2** | NM_004863.4 |
| TRPV4 | LRG_372t1 |
| TTN | LRG_3911t2 |
| TTR | LRG_416t1 |
| TRIO | NM_007118.3 |
| ZFYVE26 | NM_015346.3 |

Table 2: RefSeq– Reference Sequence; LRG – Locus Reference Genomic.A transcript’s RefSeq/LRG designation indicates that it represents a reference standard transcript curated by the NCBI RefSeq database and, where applicable, by the Locus Reference Genomic (LRG) project, which provides stable genomic sequences for clinically relevant genes.

1. Pomarino D, Emelina A, Heidrich J, et al. NGS-Panel Diagnosis Developed for the Differential Diagnosis of Idiopathic Toe Walking and Its Application for the Investigation of Possible Genetic Causes for the Gait Anomaly. Glob Med Genet. 2023;10(2):63-71. Published 2023 Apr 21. doi:10.1055/s-0043-57230
