# Supplementary material for: Toe walking: A regional analysis of TTN variants between c.10,000 and c.99,999 in a cohort of children
Source: Glob Med Genet. 2026 Aug 1;13(3):100123. doi: 10.1016/j.gmg.2026.100123 (PMC13471197; doi:10.1016/j.gmg.2026.100123)
Supplement: Supplementary file 2 [file mmc3.docx]

| **Patient ID** | **TTN variant(s)** | **Classification** | **Other gene variant(s)** | **Classification** |
| --- | --- | --- | --- | --- |
| **1** | TTN c.10018A>G | VUS | MED25 c.1004C>T | VUS |
|  | TTN c.73825G>C | VUS |  |  |
| **2** | TTN c.10045A>G | VUS | **-** |  |
| **3** | TTN c.10123G>A | VUS | TTR c.441A>T | VUS |
|  |  |  | COL6A2 c.2220_2222del | LP |
|  | TTN c.107576T>C | VUS |  |  |
|  |  |  | SBF1 c.919C>G | VUS |
|  | TTN c.15178G>C | VUS |  |  |
|  | TTN c.35385A>C | VUS | KMT2C c.-63_-52del | VUS |
| **4** | TTN c.10195T>G | VUS | CACNA1A c.*478_*483delCAGCGG | VUS |
|  | TTN c.16303G>A | VUS |  |  |
|  | TTN c.57985G>A | VUS |  |  |
| **5** | TTN c.10603G>A | VUS | SBF2 c.2323G>A | VUS |
|  | TTN c.15178G>C | VUS |  |  |
|  | TTN c.92176C>T | VUS |  |  |
| **6** | TTN c.10690G>C | VUS | ATM c.3695C>G | VUS |
|  | TTN c.33409C>T | VUS |  |  |
|  | TTN c.55619T>C | VUS | MORC2 c.*88C>T | VUS |
|  | TTN c.70579G>A | VUS |  |  |
| **7** | TTN  c.11075C>A | VUS | COL6A3 c.8212A>T | VUS |
| **8** | TTN c.11087A>G | VUS | IQSEC2 c.708-3C>T | VUS |
|  | TTN c.11672C>T | VUS |  |  |
|  | TTN c.21668G>A | VUS |  |  |
|  | TTN c.2611G>T | VUS |  |  |
|  | TTN c.31757C>A | VUS |  |  |
|  | TTN c.72379G>A | VUS |  |  |
|  | TTN c.77813G>C | VUS |  |  |
|  | TTN c.88721G>A | VUS |  |  |
| **9** | TTN c.11455C>T | VUS | ATM c.1564_1565delGA | P |
| **10** | TTN c.11635A>G | VUS | ATXN1 c.556A>T | VUS |
|  | TTN c.17129G>A | VUS | COL6A2 c.511G>A | VUS |
|  |  |  | ZFYVE26 c.1184G>T | VUS |
| **11** | TTN c.11672C>T | VUS | **-** |  |
|  | TTN c.21668G>A | VUS |  |  |
|  | TTN c.31757C>A | VUS |  |  |
|  | TTN c.59092G>T | VUS |  |  |
|  | TTN c.71642T>C | VUS |  |  |
|  | TTN c.72379G>A | VUS |  |  |
|  | TTN c.77813G>C | VUS |  |  |
|  | TTN c.88721G>A | VUS |  |  |
| **12** | TTN c.11809A>C | VUS | ATXN7 c.1958C>T | VUS |
|  | TTN c.4247G>A | VUS |  |  |
| **13** | TTN c.11996A>G | VUS | **-** | **VUS** |
|  | TTN c.52880G>A | VUS |  |  |
| **14** | TTN c.12161A>C | VUS | **-** |  |
| **15** | TTN c.12178G>A | VUS | CHRNE c.575T>C | VUS |
|  | TTN c.14450A>C | VUS | COL6A3 c.3271G>A | VUS |
|  | TTN c.34864G>A | VUS |  |  |
|  | TTN c.875C>T | VUS |  |  |
| **16** | TTN c.12205G>A | VUS | **-** |  |
| **17** | TTN c.12524C>A | VUS | PYGM c.1190T>C | **P** |
|  | TTN c.104098C>T | VUS | ATM c.4768C>T | **VUS** |
| **18** | TTN c.12667T>C | VUS | CREBBP c.2846C>T | VUS |
|  | TTN c.51569G>T | VUS | PRX c.719G>A | VUS |
| **19** | TTN c.12697G>A | VUS | **-** |  |
|  | TTN c.22190C>T | VUS |  |  |
|  | TTN c.54740T>C | VUS |  |  |
|  | TTN c.66051G>A | VUS |  |  |
| **20** | TTN c.12743A>C | VUS | CACNA1A c.6193-3C>A | VUS |
|  | TTN c.67181G>A | VUS | NDRG1 c.31G>A | VUS |
|  |  |  | PMP22 c.353C>T | P |
|  |  |  | POLG c.2481-7C>T | VUS |
|  |  |  | SBF2 c.2323G>A | VUS |
|  |  |  | ZFYVE26 c.955G>C | VUS |
| **21** | TTN c.12748G>A | VUS | CREBBP c.2728A>G | VUS |
|  | TTN c.52852C>T | VUS | MED25 c.1966C>A | LP |
|  |  |  | POLG c.970C>T | VUS |
| **22** | TTN c.12748G>A | VUS | OPA1 c.1146A>G | LP |
|  | TTN c.27025T>C | VUS |  |  |
|  | TTN c.65319T>C | VUS |  |  |
|  | TTN c.94633C>T | VUS |  |  |
| **23** | TTN c.12748G>A | VUS | DHTKD1 c.1769G>T | VUS |
|  | TTN c.52852C>T | VUS |  |  |
| **24** | TTN c.12896A>C | VUS | ALS2 c.1943A>G | VUS |
|  | TTN c.55139T>C | VUS | SH3TC2 c.385+3A>G | VUS |
| **25** | TTN c.12934C>T | VUS | CLCN1 c.2632G>A | LP |
|  |  |  | DHTKD1 c.209C>G | VUS |
| **26** | TTN c.12979A>G | VUS |  |  |
| **27** | TTN c.13123G>T | VUS | CHRNE c.45C>A | VUS |
|  | TTN c.40636C>A | VUS | NAGLU c.510C>T | VUS |
|  | TTN c.73825G>C | VUS |  |  |
| **28** | TTN c.13138T>C | VUS | ATXN2 c.-266C>T | VUS |
|  | TTN c.88394C>T | VUS | RETREG1 c.607G>A | VUS |
| **29** | TTN c.13172A>G | VUS | EGR2 c.644C>T | VUS |
|  | TTN c.69821G>A | VUS |  |  |
|  | TTN c.90403G>T | VUS |  |  |
| **30** | TTN c.13198G>T | VUS |  |  |
| **31** | TTN c.13262A>G | VUS | TRPV4 c.137C>T | VUS |
| **32** | TTN c.13262A>G | VUS | ALS2 c.2479A>T | VUS |
|  | TTN c.14870C>G | VUS |  |  |
|  | TTN c.94851T>A | VUS |  |  |
| **33** | TTN c.13376T>C | VUS | PRX c.1979C>T | VUS |
|  | TTN c.103958G>A | VUS |  |  |
| **34** | TTN c.13792C>T | VUS | TTN c.107753G>A | VUS |
|  | TTN c.30181A>C | VUS |  |  |
| **35** | TTN c.13822C>G | VUS | KMT2C c.6339A>C | VUS |
|  |  |  | PRX c.445G>A | VUS |
| **36** | TTN c.13969A>C | VUS | - | - |
|  | TTN c.63439G>A | VUS |  |  |
|  | TTN c.107576T>C | VUS |  |  |
| **37** | TTN c.14078T>C | VUS | DHTKD1 c.488G>A | VUS |
|  | TTN c.53096G>A | VUS |  |  |
|  | TTN c.67099T>C | VUS |  |  |
| **38** | TTN c.14078T>C | VUS | CLCN1 c.313C>T | P |
|  | TTN c.53096G> | VUS | CLCN1 c.501C>G | P |
|  |  |  | MORC2 c.2915G>A | VUS |
|  |  |  | POLG c.678G>C | VUS |
| **39** | TTN c.14078T>C | VUS | ATM c.185+3A>G | VUS |
|  | TTN c.53096G>A | VUS | NAGLU c.196A>T | VUS |
| **40** | TTN c.14327T>C | VUS | COL6A2 c.1097G>A | VUS |
|  | TTN c.1137A>G | VUS | MFN2 c.227T>G | VUS |
|  | TTN c.98095C>T | VUS | PMP22  duplication | P |
| **41** | TTN c.14327T>C | VUS | POLG c.391T>C | VUS |
|  | TTN c.91712T>C | VUS | PYGM c.14T>C | VUS |
| **42** | TTN c.14343G>A | VUS | SBF1 c.3191G>A | LP |
|  | TTN c.72132T>C | VUS |  |  |
| **43** | TTN c.14812A>G | VUS | COL6A3 c.898_900del | VUS |
|  | TTN c.47248G>A | VUS |  |  |
| **44** | TTN c.14870C>G | VUS | COL6A2 c.2585G>A | VUS |
|  | TTN c.94851T>A | VUS | POLG c.1964T>G | VUS |
| **45** | TTN c.14870C>G | VUS | ATM c.814A>C | VUS |
|  | TTN c.98716G>A | VUS |  |  |
| **46** | TTN c.14870C>G | VUS | COL6A3 c.2972C>T | VUS |
|  | TTN c.94851T>A | VUS | FGD4 c.2197A>G | VUS |
| **47** | TTN c.14870C>G | VUS | COL6A2 c.2608G>A | VUS |
|  | TTN c.16160G>A | VUS | PRX c.823C>A | VUS |
|  | TTN c.60721G>C | VUS |  |  |
|  | TTN c.61922G>A | VUS |  |  |
|  | TTN c.94851T>A | VUS |  |  |
|  | TTN c.98294C>G | VUS |  |  |
| **48** | TTN c.14954A>C | VUS | GDAP1 c.620A>C | VUS |
|  | TTN c.102956C>T | VUS |  |  |
| **49** | TTN c.14965C>A | VUS | - | - |
| **50** | TTN c.15011A>G | VUS | ATM c.7223C>T | VUS |
|  | TTN c.48727C>T | VUS | SH3TC2 c.79A>G | VUS |
|  | TTN c.48953T>C | VUS |  |  |
| **51** | TTN c.15056A>C | VUS | KMT2C c.925C>T | VUS |
|  | TTN c.63439G>A | VUS |  |  |
|  | TTN c.107576T>C | VUS |  |  |
| **52** | TTN c.15122C>G | VUS | COL6A2 c.900G>A | VUS |
|  | TTN c.107089G>C | VUS | RETREG1 c.607G>A | VUS |
| **53** | TTN c.15178G>C | VUS | - | - |
|  | TTN c.92176C>T | VUS |  |  |
| **54** | TTN c.15178G>C | VUS | ZFYVE26 c.6419G>A | VUS |
|  | TTN c.102713G>A | VUS | NAGLU c.1000G>A | VUS |
|  | TTN c.107576T>C | VUS |  |  |
| **55** | TTN c.15178G>C | VUS | ZFYVE26 c.5401G>A | VUS |
|  | TTN c.38437A>C | VUS | ATM c.7816A>G | VUS |
|  | TTN c.46541G>A | VUS | SBF2 c.1424A>G | VUS |
|  | TTN c.92176C>T | VUS |  |  |
| **56** | TTN c.15178G>C | VUS | - | - |
|  | TTN c.92176C>T | VUS |  |  |
|  | TTN c.98390A>G | VUS |  |  |
|  | TTN c.99025T>C | VUS |  |  |
| **57** | TTN c.15178G>C | VUS | CACNA1A c.1626G>A | VUS |
|  | TTN c.17048A>G | VUS | CLCN1 c.2680C>T | P |
|  | TTN c.50719A>G | VUS |  |  |
|  | TTN c.50719A>G | VUS |  |  |
| **58** | TTN c.15178G>C | VUS | PRX c.1216G>A | VUS |
|  | TTN c.46451G>A | VUS |  |  |
|  | TTN c.92176C>T | VUS |  |  |
| **59** | TTN c.15178G>C | VUS | CLCN1 c.854G>A | P |
|  | TTN c.38965G>A | VUS | CREBBP c.3232T>C | VUS |
|  | TTN c.92176C>T | VUS |  |  |
| **60** | TTN c.15560T>C | VUS | COL6A2 c.3025G>A | VUS |
|  | TTN c.35387C>A | VUS |  |  |
|  | TTN c.51683C>T | VUS |  |  |
| **61** | TTN c.15563A>C | VUS | KMT2C c.1336A>G | VUS |
|  |  |  | NDRG1 c.63+6T>C | VUS |
| **62** | TTN c.15563A>C | VUS | - | - |
|  | TTN c.24964G>T | VUS |  |  |
| **63** | TTN c.15563A>C | VUS | COL6A2 c.-6G>A | VUS |
|  | TTN c.24769C>G | VUS | KCNC3 c.2077A>C | VUS |
| **64** | TTN c.15563A>C | VUS | ZFYVE26 c.5320G>A | VUS |
| **65** | TTN c.15658_15661del | LP | COL6A3 c.9061G>C | VUS |
|  |  |  | MFN2 c.2119C>T | P |
|  |  |  | SBF2 c.3653-1G>A | LP |
| **66** | TTN c.15881G>T | VUS |  |  |
|  | TTN c.52702A>G | VUS |  |  |
|  | TTN c.104192A>G | VUS |  |  |
| **67** | TTN c.15986G>A | VUS | ALS2 c.1244C>T | VUS |
|  |  |  | COL6A3 c.4984G>T | VUS |
|  |  |  | KMT2C c.9245C>T | VUS |
| **68** | TTN c.15986G>A | VUS | ALS2 c.2479A>T | VUS |
|  | TTN c.83063G>A | VUS | SBF1 c.3147-8G>C | VUS |
|  |  |  | SBF1 c.4768A>G | LP |
| **69** | TTN c.16009A>G | VUS | - | - |
| **70** | TTN c.16063G>A | VUS | - | - |
|  | TTN c.49278T>C | VUS |  |  |
| **71** | TTN c.16075A>G | VUS | PYGM c.164_168del | P |
|  | TTN c.63463C>T | VUS |  |  |
| **72** | TTN c.16121G>A | VUS | NDRG1 c.31G>A | VUS |
|  |  |  | POLG c.1760C>T | P |
|  |  |  | POLG c.752C>T | P |
| **73** | TTN c.16150C>T | VUS | - | - |
|  | TTN c.72132T>C | VUS |  |  |
| **74** | TTN c.16160G>A | VUS | - | - |
|  | TTN c.64891A>G | VUS |  |  |
| **75** | TTN c.16160G>A | VUS |  |  |
|  | TTN c.60721G>C | VUS |  |  |
|  | TTN c.61922G>A | VUS |  |  |
|  | TTN c.98294C>G | VUS |  |  |
| **76** | TTN c.16160G>A | VUS | ZFYVE26 c.677G>A | VUS |
|  | TTN c.34150_34170del | VUS | CACNA1A c.6590_6601del | VUS |
|  | TTN c.98294C>G | VUS | COL6A3 c.8584G>A | VUS |
| **77** | TTN c.16160G>A | VUS | ALS2 c.2632C>T | VUS |
|  | TTN c.27847G>A | VUS | COL6A2 c.2470G>A | VUS |
|  | TTN c.32971G>A | VUS |  |  |
|  | TTN c.60721G>C | VUS |  |  |
|  | TTN c.61922G>A | VUS |  |  |
|  | TTN c.98294C>G | VUS |  |  |
| **78** | TTN c.16160G>A | VUS | SBF1 c.55G>C | VUS |
|  | TTN c.60721G>C | VUS | SH3TC2 c.101C>A | VUS |
|  | TTN c.61922G>A | VUS |  |  |
|  | TTN c.98294C>G | VUS |  |  |
| **79** | TTN c.16239G>T | VUS | COL6A3 c.4121A>T | VUS |
|  | TTN c.73825G>C | VUS |  |  |
| **80** | TTN c.16288C>T | LP | - | - |
|  | TTN c.45089_45091del | VUS |  |  |
|  | TTN c.88579A>G | VUS |  |  |
|  | TTN c.100183G>A | VUS |  |  |
| **81** | TTN c.16313A>G | VUS | - | - |
|  | TTN c.24952G>A | VUS |  |  |
|  | TTN c.76024G>C | VUS |  |  |
| **82** | TTN c.16313A>G | VUS | - | - |
|  | TTN c.53159T>C | VUS |  |  |
| **83** | TTN c.16328T>C | VUS | CLCN1 c.2680C>T | P |
|  | TTN c.45408G>T | VUS | KMT2C c.5171G> T | VUS |
| **84** | TTN c.16438G>A | VUS | CACNA1A c.6086C>T | VUS |
|  | TTN c.92176C>T | VUS |  |  |
| **85** | TTN c.16546G>T | VUS | ZFYVE26 c.4855G>A | VUS |
|  |  |  | NDRG1 c.331A>C | VUS |
|  |  |  | SBF1 c.3592C>T | VUS |
| **86** | TTN c.16546G>T | VUS | KMT2C c.7655A>G | VUS |
| **87** | TTN c.16546G>T | VUS | SH3TC2 c.1402_1403delinsTT | VUS |
|  | TTN c.22386T>A | VUS |  |  |
| **88** | TTN c.16546G>T | VUS |  |  |
|  | TTN c.18557C>T | VUS |  |  |
|  | TTN c.22386T>A | VUS |  |  |
| **89** | TTN c.16546G>T | VUS | ATM c.1564_1565del | P |
|  |  |  | SBF1 c.1312C>T | VUS |
| **90** | TTN c.16546G>T | VUS | SH3TC2 c.79A>G | VUS |
|  | TTN c.48953T>C | VUS |  |  |
| **91** | TTN c.16549T>G | VUS | COL6A2 c.2917G>T | VUS |
|  | TTN c.61115del | LP |  |  |
| **92** | TTN c.16576A>C | VUS | CHRNE c.47-8dupC | VUS |
|  | TTN c.50714G>A | VUS | PYGM c.160T>G | LP |
| **93** | TTN c.16576A>C | VUS | CHRNE c.47-8dupC | VUS |
|  | TTN c.50714G>A | VUS | PYGM c.160T>G | LP |
| **94** | TTN c.16576A>C | VUS | DHTKD1 c.128C>T | VUS |
|  | TTN c.50714G>A | VUS |  |  |
| **95** | TTN c.16613G>A | VUS | MORC2 c.2066C>T | VUS |
|  | TTN c.34307A>G | VUS | PRX c.3721G>A | VUS |
|  | TTN c.35264A>C | VUS | PYGM c.1092+6dup | VUS |
|  | TTN c.39043+2T>G | LP |  |  |
|  | TTN c.73168A>G | VUS |  |  |
|  | TTN c.97760G>C | VUS |  |  |
| **96** | TTN c.16934C>T | VUS | - |  |
|  | TTN c.41684G>A | VUS |  |  |
|  | TTN c.53096G>A | VUS |  |  |
|  | TTN c.72132T>C | VUS |  |  |
| **97** | TTN c.16934C>T | VUS | NAGLU c.2020C>T | P |
|  | TTN c.53096G>A | VUS | SBF1 c.5471G>A | VUS |
|  | TTN c.61708C>G | VUS | SBF2 c.3767_3769delTCAinsCCG | VUS |
| **98** | TTN c.17048A>G | VUS | CHRNE c.488C>T | P |
|  | TTN c.61220T>C | VUS | GARS1 c.-14A>G | VUS |
|  |  |  | PRX c.1905A>C | VUS |
| **99** | TTN c.17191T>C | VUS | ATM c.1229T>C | VUS |
|  |  |  | ATM c.5675-7_5675-4del | VUS |
|  |  |  | ATXN3 c.1043T>C | VUS |
| **100** | TTN c.17224C>T | VUS | - | - |
|  | TTN c.103688T>C | VUS |  |  |
| **101** | TTN c.17302G>A | VUS | - | - |
| **102** | TTN c.17419G>T | VUS | CLCN1 c.899G>A | VUS |
|  | TTN c.81056C>G | VUS | SBF2 c.3766G>C | VUS |
|  |  |  | TRIO c.6404T>C | VUS |
| **103** | TTN c.17818T>C | VUS | CACNA1A c.6650_6661delACCACCACCATC | VUS |
|  | TTN c.6353T>C | VUS |  |  |
| **104** | TTN c.17818T>C | VUS | PRX c.1216G>A | VUS |
| **105** | TTN c.17818T>C | VUS | COL6A3 c.4156G>A | VUS |
| **106** | TTN c.17818T>C | VUS |  |  |
|  | TTN c.69130C>T | VUS |  |  |
| **107** | TTN c.17871A>T | VUS | CAV3 c.-37G>A | VUS |
|  | TTN c.89426G>A | VUS | COL6A3 c.4156G>A | VUS |
| **108** | TTN  c.18325A>G | VUS | ZFYVE26 c.3722G>A | VUS |
|  | TTN c.32624C>T | VUS | CLCN1 c.313C>T | P |
|  | TTN c.95582A>G | VUS | CLCN1 c.501C>G | P |
|  |  |  | POLG c.1760C>T | P |
|  |  |  | POLG c.752C>T | P |
| **109** | TTN c.18368C>A | VUS | FGD4 c.-245-29728T>C | VUS |
|  |  |  | MED25 c.1004C>T | VUS |
|  |  |  | POLG c.803G>C | VUS |
| **110** | TTN c.18776C>G | VUS | PYGM c.1094C>T | P |
|  | TTN c.47315G>A | VUS |  |  |
|  | TTN c.80701A>G | VUS |  |  |
|  | TTN c.85462G>A | VUS |  |  |
| **111** | TTN c.18776C>G | VUS | - | - |
|  | TTN c.28131C>T | VUS |  |  |
|  | TTN c.47315G>A | VUS |  |  |
| **112** | TTN c.18776C>G | VUS | ZFYVE26 c.1184G>T | VUS |
|  | TTN c.47315G>A | VUS | COL6A2 c.2917G>A | VUS |
| **113** | TTN c.18776C>G | VUS | CACNA1A c.3043G>A | VUS |
|  | TTN c.72232A>G | VUS |  |  |
| **114** | TTN c.18776C>G | VUS | CLCN1 c.1393G>A | VUS |
|  | TTN c.47315G>A | VUS | OPA1 c.113_130del | LP |
|  | TTN c.54541G>C | VUS | POLG c.2481-7C>T | VUS |
|  | TTN c.74305A>G | VUS |  |  |
| **115** | TTN c.18920G>C | VUS | COL6A3 c.4156G>A | LP |
|  |  |  | COL6A3 c.9234del | LP |
|  |  |  | DHTKD1 c.1315G>C | VUS |
|  |  |  | POLG c.803G>C | VUS |
| **116** | TTN c.19150C>A | VUS | NDRG1 c.331A>C | VUS |
|  | TTN c.63589A>G | VUS |  |  |
| **117** | TTN c.19150C>A | VUS | MORC2 c.2929G>A | VUS |
|  | TTN c.86234C>G | VUS | PMP22 c.353C>T | LP |
| **118** | TTN c.20156T>C | VUS | ZFYVE26 c.6940A>C | VUS |
|  | TTN c.68458G>C | VUS | ATXN1 c.510G>C | VUS |
|  | TTN c.88135G>C | VUS |  |  |
| **119** | TTN c.20341G>A | VUS | MFN2 c.1403G>A | LP |
|  | TTN c.26494A>G | VUS |  |  |
|  | TTN c.94846C>T | VUS |  |  |
| **120** | TTN c.20341G>A | VUS | ALS2 c.4119A>G | VUS |
|  | TTN c.26494A>G | VUS |  |  |
|  | TTN c.94846C>T | VUS |  |  |
| **121** | TTN c.20341G>A | VUS | - | - |
|  | TTN c.94846C>T | VUS |  |  |
| **122** | TTN c.20341G>A | VUS | - | - |
|  | TTN c.26494A>G | VUS |  |  |
|  | TTN c.94846C>T | VUS |  |  |
| **123** | TTN c.20341G>A | VUS | ZFYVE26 c.2300G>A | VUS |
|  | TTN c.26494A>G | VUS | ATM c.6067G>A | VUS |
|  | TTN c.94846C>T | VUS | CREBBP c.2728A>G | VUS |
|  |  |  | DHTKD1 c.2530G>T | VUS |
| **124** | TTN c.20354C>T | VUS | PYGM c.645G>A | VUS |
|  | TTN c.31762+5G>A | VUS |  |  |
|  | TTN c.87280G>A | VUS |  |  |
| **125** | TTN c.20355G>A | VUS | CREBBP c.5837C>T | VUS |
|  | TTN c.32624C>T | VUS | POLG c.970C>T | VUS |
| **126** | TTN c.20395C>T | LP | COL6A2 c.511G>A | VUS |
|  | TTN c.72146T>C | VUS |  |  |
| **127** | TTN c.20418A>C | VUS | POLG c.c.1550G>T | VUS |
|  | TTN c.48953T>C | VUS |  |  |
|  | TTN c.70111C>T | VUS |  |  |
| **128** | TTN c.21002A>G | VUS | COL6A3 c.1613C>T | VUS |
|  | TTN c.49871G>A | VUS | KMT2C c.13311G>A | VUS |
| **129** | TTN c.21157A>C | VUS | TRIO c.7397C>T | VUS |
|  | TTN c.94045C>T | VUS |  |  |
| **130** | TTN c.21364G>A | VUS | - | - |
|  | TTN c.30718G>T | VUS |  |  |
| **131** | TTN c.21364G>A | VUS | COL6A2 c.511G>A | VUS |
|  | TTN c.39466C>A | VUS |  |  |
| **132** | TTN c.21364G>A | VUS | - | - |
| **133** | TTN c.21364G>A | VUS | FBLN5 c.1223G>A | VUS |
|  |  |  | PYGM c.577G>T | VUS |
| **134** | TTN c.21404-4A>G | VUS | ZFYVE26 c.827G>A | VUS |
|  | TTN c.23378-10C>A | VUS | COL6A3 c.7114G>A | VUS |
|  | TTN c.59113C>T | VUS |  |  |
| **135** | TTN c.21404-4A>G | VUS | KMT2C c.11719A>G | VUS |
|  | TTN c.23378-10C>A | VUS |  |  |
|  | TTN c.59113C>T | VUS |  |  |
| **136** | TTN c.21555C>A | VUS |  |  |
|  | TTN c.33796C>T | VUS |  |  |
|  | TTN c.91557T>C | VUS |  |  |
| **137** | TTN c.21656C>T | VUS | ZFYVE26 c.1184G>T | VUS |
|  |  |  | CLCN1 c.501C>G | LP |
|  |  |  | COL6A3 c.424G>T | VUS |
|  |  |  | RETREG1 c.1451A>G | VUS |
|  |  |  | SBF1 c.4708C>T | VUS |
| **138** | TTN c.21656C>T | VUS | CACNA1A c.3043G>A | VUS |
|  | TTN c.43986T>G | VUS | EPHB4 c.1561G>A | VUS |
|  | TTN c.73847G>A | VUS |  |  |
| **139** | TTN c.21656C>T | VUS |  |  |
| **140** | TTN c.21682+5G>C | LP | CACNA1A c.*772_*780del, | VUS |
|  | TTN c.23291T>C | VUS | SH3TC2 c.3686A>T | VUS |
|  | TTN c.28894C>T | VUS |  |  |
|  | TTN c.41407G>A | VUS |  |  |
| **141** | TTN c.21712G>A | VUS | COL6A3 c.4541T>C | VUS |
|  | TTN c.60407T>A | VUS |  |  |
| **142** | TTN c.22439A>C | VUS | ZFYVE26 c.878C>T | VUS |
|  |  |  | KMT2C c.6339A>C | VUS |
| **143** | TTN c.22439A>C | VUS | RETREG1 c.607G>A | VUS |
|  | TTN c.3608G>C | VUS |  |  |
|  | TTN c.1137A>G | VUS |  |  |
| **144** | TTN c.22531C>T | VUS |  |  |
|  | TTN c.72132T>C | VUS |  |  |
| **145** | TTN c. 22634G>A | VUS | COL6A3 c.489G>A | VUS |
|  |  |  | SH3TC2 c.581A>G | VUS |
| **146** | TTN c.23302G>A | VUS |  |  |
| **147** | TTN c.23302G>A | VUS |  |  |
|  | TTN c.32624C>T | VUS |  |  |
|  | TTN c.36391C>T | VUS |  |  |
| **148** | TTN c.23302G>A | VUS | CAV3 c.276C>T | VUS |
|  |  |  | COL6A3 c.6174C>T | VUS |
|  |  |  | FXN c.384+8T>C | VUS |
|  |  |  | SH3TC2 c.1318C>T | VUS |
| **149** | TTN c.23302G>A | VUS | COL6A2 c.791G>A | VUS |
|  |  |  | FXN c.118C>T | VUS |
| **150** | TTN c.23308G>A | VUS | COL6A2 c.2795C>T | VUS |
|  |  |  | POLG c.1760C>T | P |
|  |  |  | POLG c.752C>T | P |
|  |  |  | TRIO c.8716C>A | VUS |
| **151** | TTN c.23353T>C | VUS |  |  |
|  | TTN c.7711G>A | VUS |  |  |
| **152** | TTN c.24371G>A | VUS |  |  |
| **153** | TTN c.24622G>A | VUS | SBF2 c.-47C>T | VUS |
| **154** | TTN c.24964G>T | VUS | EGR2 c.733G>A | VUS |
|  |  |  | OPA1 c.344C>T | VUS |
| **155** | TTN c.24964G>T | VUS |  |  |
| **156** | TTN c.25126C>T | VUS | ATXN7 c.2119C>A | VUS |
|  |  |  | CREBBP c.5800T>C | VUS |
| **157** | TTN c.25126C>T | VUS |  |  |
|  | TTN c.105490C>T | VUS |  |  |
| **158** | TTN c.25126C>T | VUS | ATXN7 c.2119C>A | VUS |
|  |  |  | CREBBP c.5800T>C | VUS |
| **159** | TTN c.25850G>A | VUS | ALS2 c.1115C>G | VUS |
|  | TTN c.31694A>G | VUS |  |  |
|  | TTN c.48164G>A | VUS |  |  |
| **160** | TTN c.26134G>C | VUS | ATM c.7865C>T | P |
|  |  |  | POLG c.2481-7C>T | VUS |
| **161** | TTN c.26695G>T | VUS |  |  |
|  | TTN c.34150_34170del | VUS |  |  |
|  | TTN c.98294C>G | VUS |  |  |
|  |  |  |  |  |
| **162** | TTN c.26792A>T | VUS |  |  |
|  | TTN c.68458G>C | VUS |  |  |
|  | TTN c.73994C>T | VUS |  |  |
|  | TTN c.83618T>C | VUS |  |  |
|  | TTN c.1301A>G | VUS |  |  |
| **163** | TTN c.26863A>G | VUS |  |  |
|  | TTN c.103147G>C | VUS |  |  |
| **164** | TTN c.27815G>C | VUS | ALS2 c.1589C>G | VUS |
| **165** | TTN c.27815G>C | VUS | COL6A3 c.4156G>A | VUS |
| **166** | TTN c.27847G>A | VUS | COL6A2 c.511G>A | VUS |
| **167** | TTN c.28058G>A | VUS |  |  |
| **168** | TTN c.28465C>T | VUS | CAV3 c.276C>T | VUS |
|  | TTN c.88282C>T | VUS |  |  |
| **169** | TTN c.28733C>T | VUS | DHTKD1 c.2185G>A | P |
|  | TTN c.30485C>T | VUS | SH3TC2 c.1298C>T | VUS |
|  | TTN c.62572A>G | VUS |  |  |
|  | TTN c.88394C>T | VUS |  |  |
|  | TTN c.92806G>A | VUS |  |  |
| **170** | TTN c.29219A>G | VUS | ATXN7 c.1964C>G | VUS |
|  | TTN c.56693G>A | VUS | NEFL c.639C>G | VUS |
|  | TTN c.93005G>T | VUS |  |  |
| **171** | TTN c.29230C>T | VUS | POLG c.2481-7C>T | VUS |
|  |  |  | SH3TC2 c.3380G>A | LP |
| **172** | TTN c.29870C>T | VUS |  |  |
| **173** | TTN c.29964G>T | VUS | ALS2 c.1677A>G | VUS |
|  | TTN c.71113C>G | VUS | PRX c.1216G>A | VUS |
|  |  |  | SBF1 c.3044G>A | VUS |
| **174** | TTN c.30224-3delC | VUS |  |  |
|  | TTN c.30389G>A | VUS |  |  |
|  | TTN c.48953T>C | VUS |  |  |
|  | TTN c.100432T>G | VUS |  |  |
|  | TTN c.100447G>C | VUS |  |  |
| **175** | TTN c.30274C>T | VUS | ATXN2 c.2520A>G | VUS |
|  | TTN c.39115A>C | VUS |  |  |
|  | TTN c.98242C>T | VUS |  |  |
|  | TTN c.104640G>A | VUS |  |  |
| **176** | TTN c.30389G>A | VUS | ZFYVE26 c.6154G>A | VUS |
|  | TTN c.41403C>A | VUS |  |  |
|  | TTN c.100432T>G | VUS |  |  |
|  | TTN c.100447G>C | VUS |  |  |
| **177** | TTN c.30389G>A | VUS | GDAP1 c.715C>T | P |
|  | TTN c.51443C>G | VUS |  |  |
|  | TTN c.100432T>G | VUS |  |  |
| **178** | TTN c.30389G>A | VUS |  |  |
|  | TTN c.45508G>A | VUS |  |  |
|  | TTN c.100432T>G | VUS |  |  |
|  | TTN c.100447G>C | VUS |  |  |
| **179** | TTN c.30485C>T | VUS |  |  |
|  | TTN c.62572A>G | VUS |  |  |
|  | TTN c.3002T>G | VUS |  |  |
| **180** | TTN c.30513A>T | VUS | COL6A3 c.3685G>A | VUS |
|  | TTN c.47248G>A | VUS | POLG c.803G>C | VUS |
|  |  |  | SBF1 c.3922C>T | VUS |
| **181** | TTN c.30683-2A>T | VUS | COL6A2 c.2585G>A | VUS |
|  | TTN c.68786C>T | VUS | IQSEC2 c.684C>T | VUS |
| **182** | TTN c.30718G>T | VUS | POLG c.1550G>T | VUS |
| **183** | TTN c.30718G>T | VUS | ATXN3 c.916_917insAGCAGCAGCAGCAGCAGC | VUS |
| **184** | TTN c.30718G>T | VUS | CLCN1 c.2680C>T | P |
| **185** | TTN c.30718G>T | VUS | ATXN7 c.1199G>A | VUS |
|  | TTN c.47248G>A | VUS | CHRNE c.901G>A | VUS |
|  |  |  | DHTKD1 c.2326G>A | VUS |
| **186** | TTN c.30718G>T | VUS | CAV3 c.-37G>A | VUS |
|  |  |  | COL6A2 c.2170C>T | VUS |
|  |  |  | SBF2 c.2323G>A | VUS |
|  |  |  | SBF2 c.4634T>C | VUS |
| **187** | **TTN c.31399G>A** | **VUS** |  |  |
| **188** | TTN c.31399G>A | VUS | CLCN1 c.2680C>T | P |
|  | TTN c.63589A>G | VUS |  |  |
| **189** | TTN c.31757C>A | VUS |  |  |
|  | TTN c.38455G>T | VUS |  |  |
|  | TTN c.72379G>A | VUS |  |  |
|  | TTN c.77813G>C | VUS |  |  |
|  | TTN c.88721G>A | VUS |  |  |
| **190** | TTN c.31762+5_31762+7del | VUS |  |  |
|  | TTN c.32624C>T | VUS |  |  |
|  | TTN c.47191C>T | VUS |  |  |
| **191** | TTN c.31763-1G>A | LP |  |  |
|  | TTN c.51809G>T | VUS |  |  |
| **192** | TTN c.31763-1G>A | LP | COL6A3 c.1975C>T | VUS |
|  | TTN c.76483G>A | VUS | CREBBP c.6463C>T | VUS |
| **193** | TTN c.31807G>A | VUS | PYGM c.1888G>A | VUS |
|  | TTN c.3619C>A | VUS | TTR c.424G>A | P |
|  | TTN c.44077C>T | VUS |  |  |
|  | TTN c.7961G>A | VUS |  |  |
| **194** | TTN c.31837C>G | VUS | ZFYVE26 c.*42G>C | VUS |
|  |  |  | ATXN1 c.1563G>C | VUS |
|  |  |  | CREBBP c.69G>A | VUS |
| **195** | TTN c.31837C>G | VUS | ZFYVE26 c.1184G>T | VUS |
|  |  |  | MORC2 c.*22C>T | VUS |
|  |  |  | SH3TC2 c.2582G>A | VUS |
| **196** | TTN c.31837C>G | VUS | ALS2 c.1115C>G | VUS |
|  |  |  | ATM c.5005+7_5005+8del | VUS |
| **197** | TTN c.31873C>G | VUS |  |  |
|  | TTN c.7060C>T | VUS |  |  |
| **198** | TTN c.31873C>G | VUS | COL6A3 c.776C>T | VUS |
|  |  |  | POLG c.*63A>G | VUS |
|  |  |  | POLG c.3131T>C | VUS |
|  |  |  | SH3TC2 c.1402_1403delinsTT | VUS |
|  |  |  | ZFYVE26 c.3587T>G | VUS |
| **199** | TTN c.31875A>C | VUS | COL6A2 c.2707G>A | VUS |
|  | TTN c.62385C>A | VUS |  |  |
| **200** | TTN c.31880A>G | VUS | COL6A3 c.6068A>G | VUS |
|  | TTN c.69130C>T | VUS | PRX c.2463C>T | VUS |
|  | TTN c.79315C>T | VUS |  |  |
| **201** | TTN c.32240G>A | VUS |  |  |
| **202** | TTN c.32462C>T | VUS | COL6A2 c.2371G>A | VUS |
|  | TTN c.83936A>G | VUS |  |  |
| **203** | TTN c.32557C>T | VUS | CHRNE c.465C>T | VUS |
|  |  |  | SBF1 c.4820G>A | VUS |
| **204** | TTN c.32624C>T | VUS | SH3TC2 c.505T>C | VUS |
|  | TTN c.48727C>T | VUS |  |  |
| **205** | TTN c.32624C>T | VUS | DHTKD1 c.923G>A | VUS |
|  |  |  | FGD4 c.479G>A | VUS |
|  |  |  | FXN c.483T>C | VUS |
| **206** | TTN c.32624C>T | VUS | MED25 c.1004C>T | VUS |
|  |  |  | PMP22 c.*3C>T | VUS |
|  |  |  | POLG c.803G>C | VUS |
| **207** | TTN c.32624C>T | VUS |  |  |
|  | TTN c.36391C>T | VUS |  |  |
|  | TTN c.81038G>A | VUS |  |  |
| **208** | TTN c.32624C>T | VUS | ATXN1 c.313G>A | VUS |
|  | TTN c.47191C>T | VUS |  |  |
| **209** | TTN c.32624C>T | VUS |  |  |
|  | TTN c.67975G>A | VUS |  |  |
|  | TTN c.69821G>A | VUS |  |  |
| **210** | TTN c.32624C>T | VUS | COL6A3 c.6898G>A | VUS |
|  | TTN c.47191C>T | VUS | COL6A3 c.9061_9063del | LP |
|  |  |  | KMT2C c.925C>T | VUS |
|  |  |  | PYGM c.660G>A | VUS |
| **211** | TTN c.32624C>T | VUS |  |  |
|  | TTN c.47191C>T | VUS |  |  |
| **212** | TTN c.32624C>T | VUS | COL6A3 c.8825C>T | VUS |
|  | TTN c.53780T>C | VUS |  |  |
| **213** | TTN c.32624C>T | VUS | SH3TC2 c.1006A>G | VUS |
| **214** | TTN c.32624C>T | VUS | COL6A2 c.1828C>T | VUS |
|  |  |  | SBF2 c.2100+4A>G | VUS |
| **215** | TTN c.32624C>T | VUS | FXN c.13G>C | VUS |
|  | TTN c.47191C>T | VUS | SBF1 c.1891_1892delinsGA | VUS |
| **216** | TTN c.32624C>T | VUS | ATXN7 c.1403T>C | VUS |
|  | TTN c.47191C>T | VUS | CHRNE c.1326+6A>C | VUS |
| **217** | TTN c.32624C>T | VUS |  |  |
| **218** | TTN c.32891T>C | VUS | NAGLU c.934G>A | P |
|  |  |  | TTN c.14050G>A | VUS |
| **219** | TTN c.33247G>C | VUS | ZFYVE26 c.3019+3G>T | VUS |
|  |  |  | ATM c.4279G>A | VUS |
|  |  |  | GDAP1 c.841G>A | VUS |
| **220** | TTN c.33305G>A | VUS | PYGM c.660G>A | VUS |
|  |  |  | SH3TC2 c.1942C>T | VUS |
| **221** | TTN c.33305G>A | VUS | ATM c.6067G>A | VUS |
|  |  |  | CREBBP c.2811G>A | VUS |
|  |  |  | LITAF c.381G>C | VUS |
|  |  |  | SH3TC2 c.1942C>T | VUS |
| **222** | TTN c.33328C>T | VUS | COL6A2 c.2580G>A | VUS |
|  | TTN c.57442A>G | VUS |  |  |
| **223** | TTN c.33328C>T | VUS | CREBBP c.2728A>G | VUS |
|  | TTN c.57442A>G | VUS |  |  |
| **224** | TTN c.33377A>C | VUS |  |  |
|  | TTN c.55925T>A | VUS |  |  |
|  | TTN c.64916G>A | VUS |  |  |
| **225** | TTN c.33856G>A | VUS |  |  |
| **226** | TTN c.33856G>A | VUS | AIFM1 c.1378G>A | VUS |
|  | TTN c.97331G>C | VUS |  |  |
| **227** | TTN c.33911-12_33911-10del | VUS | POLG c.3442C>T | VUS |
| **228** | TTN c.33920T>G | VUS | ALS2 c.3860_3865del | VUS |
|  | TTN c.49413G>T | VUS | ATXN7 c.2159C>G | VUS |
|  | TTN c.98390A>G | VUS | COL6A3 c.6255G>A | VUS |
|  |  |  | GARS1 c.764C>T | VUS |
| **229** | TTN c.34094C>T | VUS | ALS2 c.1834G>T | VUS |
|  |  |  | NEFL c.400C>T | LP |
| **230** | TTN c.34119_34139dup | VUS |  |  |
|  | TTN c.88394C>T | VUS |  |  |
| **231** | TTN c.34150_34170del | VUS |  |  |
|  | TTN c.98294C>G | VUS |  |  |
| **232** | TTN c.34216C>A | VUS | PRX c.3373G>A | VUS |
| **233** | TTN c.34247_34249del | VUS | CLCN1 c.442T>C | LP |
|  | TTN c.63439G>A | VUS |  |  |
|  | TTN c.107576T>C | VUS |  |  |
| **234** | TTN c.34247_34249del | VUS | COL6A3 c.5387C>T | VUS |
|  |  |  | PYGM c.660G>A | VUS |
| **235** | TTN c.34247_34249del | VUS | ATM c.146C>G | VUS |
|  |  |  | ATM c.7375C>T | VUS |
| **236** | TTN c.34601T>C | VUS |  |  |
|  | TTN c.7891G>A | VUS |  |  |
| **237** | TTN c.34864G>A | VUS | ATXN2 c.3228G>A | VUS |
|  |  |  | FGD4 c.1366C>A | VUS |
|  |  |  | MORC2 c.2313G>A | VUS |
|  |  |  | TRIO c.3763T>G | VUS |
| **238** | TTN c.34864G>A | VUS | COL6A2 c.2875G>A | VUS |
|  |  |  | SBF1 c.3922C>T | VUS |
| **239** | TTN c.34864G>A | VUS | SBF1 c.1783C>T | LP |
| **240** | TTN c.34864G>A | VUS | POLG c.328C>T | VUS |
| **241** | TTN c.35387C>A | VUS | POLG c.629C>T | VUS |
|  | TTN c.51683C>T | VUS | SBF2 c.5245A>G | VUS |
|  |  |  | SH3TC2 c.3380G>A | VUS |
| **242** | TTN c.35387C>A | VUS | SBF1 c.2738C>T | VUS |
|  | TTN c.51683C>T | VUS | SBF1 c.3958G>A | VUS |
|  | TTN c.69821G>A | VUS | SBF1 c.3985G>A | VUS |
| **243** | TTN c.36044C>G | VUS | COL6A3 c.4626A>G | VUS |
|  | TTN c.63877G>A | VUS | DHTKD1 c.209C>G | VUS |
|  | TTN c.74921A>C | VUS | GARS1 c.1905G>A | VUS |
|  | TTN c.13412G>C | VUS | MORC2 c.*88C>T | VUS |
|  |  |  | POLG c.2481-7C>T | VUS |
| **244** | TTN c.36044C>G | VUS | COL6A3 c.4626A>G | VUS |
|  | TTN c.74921A>C | VUS | GARS1 c.1905G>A | VUS |
|  |  |  | POLG c.2481-7C>T | VUS |
| **245** | TTN c.38297-9T>A | VUS | MED25 c.1762C>T | VUS |
| **246** | TTN c.38297-9T>A | VUS | ZFYVE26 c.2551C>A | VUS |
| **247** | TTN c.38361G>A | VUS | ATXN1 c.556A>T | VUS |
|  |  |  | CACNA1A c.1626G> | VUS |
|  |  |  | COL6A3 c.9013G>A | VUS |
|  |  |  | EPHB4 c.236C>T | VUS |
| **248** | TTN c.38371C>T | VUS |  |  |
|  | TTN c.90994G>A | VUS |  |  |
| **249** | TTN c.39044-9T>A | VUS |  |  |
|  | TTN c.99162G>A | VUS |  |  |
| **250** | TTN c.39044-9T>A | VUS | TRIO c.7268C>T | VUS |
|  | TTN c.72146T>C | VUS |  |  |
|  | TTN c.99162G>A | VUS |  |  |
| **251** | TTN c.39115A>C | VUS | PRX c.3707C>T | VUS |
| **252** | TTN c.39115A>C | VUS | CREBBP c.3604C>T | VUS |
|  | TTN c.65850G>A | VUS |  |  |
|  | TTN c.92780T>A | VUS |  |  |
| **253** | TTN c.39115A>C | VUS | ATXN2 c.1526C>T | VUS |
|  |  |  | SBF1 c.410C>T | VUS |
|  |  |  | SH3TC2 c.645C>T | VUS |
| **254** | TTN c.39115A>C | VUS | ATXN3 c.891_905del | VUS |
|  |  |  | CREBBP c.712G>A | VUS |
|  |  |  | GARS1 c.1852G>A | VUS |
| **255** | TTN c.39115A>C | VUS | CACNA1A c.3787G>A | VUS |
|  | TTN c.69130C>T | VUS | SBF1 c.1364C>T | VUS |
|  |  |  | SBF2 c.2197C>G | VUS |
| **256** | TTN c.39220G>T | VUS |  |  |
|  | TTN c.103302T>C | VUS |  |  |
| **257** | TTN c.39466C>A | VUS | TRIO c.7379C>T | VUS |
| **258** | TTN c.39466C>A | VUS | CREBBP c.5507A>G | VUS |
|  | TTN c.91616A>G | VUS | SPTLC2 c.117_122dup | VUS |
| **259** | TTN c.40023_40029del | P | - | - |
| **260** | TTN c.40595T>C | VUS | POLG c.2467C>T | VUS |
|  | TTN c.98075C>G | VUS |  |  |
| **261** | TTN c.40636C>A | VUS |  |  |
|  | TTN c.101282G>A | VUS |  |  |
| **262** | TTN c.40636C>A | VUS |  |  |
| **263** | TTN c.40842C>T | VUS | ATM c.2572T>C | VUS |
|  | TTN c.48903G>C | VUS | ATXN2 c.2326A>G | VUS |
|  | TTN c.72132T>C | VUS | COL6A3 c.4156G>A | VUS |
|  |  |  | IQSEC2 c.3836C>T | VUS |
| **264** | TTN c.41230C>T | VUS | ATM c.146C>G | VUS |
|  | TTN c.62385C>A | VUS | POLG c.803G>C | VUS |
| **265** | TTN c.41309C>T | VUS | PYGM c.1094C>T | P |
| **266** | TTN c.41330-7T>A | VUS | DHTKD1 c.1364G>A | VUS |
|  | TTN c.83516G>A | VUS | TRIO c.3066+5G>A | VUS |
| **267** | TTN c.41467C>G | VUS | POLG c.1550G>T | VUS |
| **268** | TTN c.41684G>A | VUS |  |  |
|  | TTN c.72132T>C | VUS |  |  |
| **269** | **TTN c.41686G>T** | **VUS** | CREBBP c.6728C>T | VUS |
|  | TTN c.50714G>A | VUS | NAGLU c.1811C>T | P |
|  |  |  | PYGM c.645G>A | VUS |
| **270** | **TTN c.41716T>C** | **VUS** |  |  |
|  | TTN c.57860G>A | VUS |  |  |
|  | TTN c.61448G>A | VUS |  |  |
| **271** | TTN c.42023C>T | VUS |  |  |
|  | TTN c.2354T>A | VUS |  |  |
| **272** | TTN c.42046G>C | VUS | KMT2C c.14074G>A | VUS |
|  |  |  | MORC2 c.712C>T | VUS |
| **273** | TTN c.42215G>A | VUS | ZFYVE26 c.7128+4C>T | VUS |
| **274** | TTN c.42329T>C | VUS | ATM c.6067G>A | VUS |
|  | TTN c.5993G>A | VUS | COL6A3 c.2972C>T | VUS |
|  | TTN c.1137A>G | VUS |  |  |
| **275** | TTN c.42847C>T | VUS | COL6A2 c.2170C>T | VUS |
|  |  |  | RETREG1 c.607G>A | VUS |
| **276** | TTN c.42847C>T | VUS |  |  |
| **277** | TTN c.43138T>C | VUS |  |  |
| **278** | TTN c.43138T>C | VUS | COL6A3 c.4529G>C | VUS |
| **279** | TTN c.43138T>C | VUS | EPHB4 c.1085G>A | VUS |
|  |  |  | KMT2C c.11443A>G | VUS |
| **280** | TTN c.43435T>A | VUS | KCNC3 c.1746_1754del | LP |
|  | TTN c.75974G>A | LP |  |  |
|  | TTN c.106996C>G | VUS |  |  |
| **281** | TTN c.43478A>G | VUS | PYGM c.1094C>T | LP |
|  |  |  | SBF1 c.5471G>A | VUS |
| **282** | TTN c.44005G>A | VUS |  |  |
| **283** | TTN c.44530G>A | VUS | COL6A3 c.1397A>G | VUS |
|  | TTN c.68458G>C | VUS | ZFYVE26 c.7417-5G>C | VUS |
|  |  |  | COL6A3 c.9508G>A | VUS |
| **284** | TTN c.45199G>A | VUS | COL6A3 c.4156G>A | VUS |
| 285 | TTN c.45268C>A | VUS | ATM c.3040G>C | VUS |
|  |  |  | ATM c.4152T>A | VUS |
| **286** | TTN c.45408G>T | VUS | SH3TC2 c.1402_1403delinsTT | VUS |
|  | TTN c.6227A>G | VUS |  |  |
|  | TTN c.7468C>T | VUS |  |  |
| **287** | TTN c.45408G>T | VUS |  |  |
| 288 | TTN c.45408G>T | VUS | MPZ c.476G>C | LP |
| 289 | TTN c.45408G>T | VUS | ATM c.26T>A | VUS |
|  | TTN c.52966G>A | VUS | CACNA1A c.5959A>G | VUS |
|  | TTN c.64457T>A | VUS | GARS1 c.1429A>G | VUS |
|  | TTN c.64465C>T | VUS | SBF1 c.1312C>T | VUS |
|  | TTN c.2536T>G | VUS |  |  |
| 290 | TTN c.45408G>T | VUS |  |  |
|  | TTN c.78405G>C | VUS |  |  |
| 291 | TTN c.45408G>T | VUS | SBF2 c.2323G>A | VUS |
| 292 | TTN c.45408G>T | VUS | CREBBP c.5837C>A | VUS |
|  | TTN c.73825G>C | VUS | KMT2C c.6339A>C | VUS |
| 293 | TTN c.45408G>T | VUS | ALS2 c.3658A>G | VUS |
|  |  |  | ZFYVE26 c.3722G>A | VUS |
| 294 | TTN c.45408G>T | VUS | CACNA1A c.5989A>G | VUS |
|  | TTN c.98726T>C | VUS |  |  |
| 295 | TTN c.45979C>T | VUS |  |  |
|  | TTN c.105514_105516del | VUS |  |  |
|  | TTN c.69821G>A | VUS |  |  |
| 296 | TTN c.45979C>T | VUS | CACNA1A c.6517G>T | VUS |
|  | TTN c.69821G>A | VUS | MFN2 c.2119C>T | P |
|  | TTN c.105514_105516del | VUS |  |  |
| 297 | TTN c.45979C>T | VUS | CACNA1A c.2896G>A | VUS |
|  | TTN c.69821G>A | VUS |  |  |
|  | TTN c.105514_105516del | VUS |  |  |
| 298 | TTN c.46354T>G | VUS | ZFYVE26 c.7417-5G>C | VUS |
| 299 | TTN c.46757dupC | LP | SBF1 c.-21_-3del, | VUS |
|  | TTN c.68458G>C | VUS | SBF1 c.3808G>A | VUS |
|  | TTN c.69821G>A | VUS |  |  |
| 300 | TTN c.47089C>T | VUS | CLCN1 c.2545G>A | VUS |
|  |  |  | KMT2C c.2776A>G | VUS |
| **301** | TTN c.47248G>A | VUS |  |  |
|  | TTN c.88394C>T | VUS |  |  |
| **302** | **TTN c.47248G>A** | **VUS** | ATM c.6820G>A | VUS |
|  | TTN c.415C>T | VUS | EGR2 c.1277G>A | VUS |
|  |  |  | KMT2C c.6339A>C | VUS |
|  |  |  | POLG c.2481-7C>T | VUS |
| **303** | TTN c.47315G>A | VUS |  |  |
| **304** | TTN c.48727C>T | VUS | SBF2 c.4394G>A | VUS |
| **305** | TTN c.48727C>T | VUS | SH3TC2 c.3795G>C | VUS |
| **306** | TTN c.48727C>T | VUS | PYGM c.1537A>G | VUS |
|  |  |  | SH3TC2 c.3795G>C | VUS |
| **307** | TTN c.48727C>T | VUS | TTR c.360C>T | VUS |
|  |  |  | ATXN2 c.1805A>T | VUS |
|  |  |  | ATXN7 c.978C>T | VUS |
|  |  |  | KMT2C c.5512C>T | VUS |
| **308** | TTN c.48727C>T | VUS | POLG c.1550G>T | VUS |
| **309** | TTN c.48727C>T | VUS | SBF2 c.4054A>G | VUS |
|  |  |  | SH3TC2 c.3795G>C | VUS |
| **310** | TTN c.48727C>T | VUS | CREBBP c.2728A>G | VUS |
|  | TTN c.93616A>C | VUS |  |  |
| **311** | TTN c.48727C>T | VUS | SBF1 c.2663G>A | VUS |
| **312** | TTN c.48727C>T | VUS | ALS2 c.4670A>G | LP |
|  |  |  | ATM c.4324T>C | VUS |
|  |  |  | FBLN5 c.799G>A | VUS |
|  |  |  | KMT2C c.12401C>T | VUS |
|  |  |  | POLG c.803G>C | VUS |
| **313** | TTN c.48727C>T | VUS |  |  |
| **314** | TTN c.48727C>T | VUS |  |  |
| **315** | TTN c.48727C>T | VUS |  |  |
| **316** | TTN c.48727C>T | VUS |  |  |
| **317** | TTN c.48727C>T | VUS | NEFL c.639C>G | VUS |
| **318** | TTN c.48727C>T | VUS | COL6A2 c.2998A>G | VUS |
| **319** | TTN c.48727C>T | VUS | PYGM c.660G>A | VUS |
| **320** | TTN c.48953T>C | VUS |  |  |
|  | TTN c.102329G>A | VUS |  |  |
| **321** | TTN c.49244T>C | VUS | SBF1 c.3157C>T | VUS |
|  |  |  | TRIO c.7453T>C | VUS |
| **322** | TTN c.49413G>T | VUS | PRX c.1216G>A | VUS |
|  |  |  | PYGM c.645G>A | VUS |
| **323** | TTN c.49593T>C | VUS |  |  |
|  | COL6A3 c.4156G>A | VUS |  |  |
|  | GARS1 c.-19A>C | VUS |  |  |
| **324** | TTN c.50072G>C | VUS | ZFYVE26 c.1184G>T | VUS |
|  | TTN c.74600_74602delCAA | VUS |  |  |
|  | TTN c.94553T>C | VUS |  |  |
|  | TTN c.104005G>A | VUS |  |  |
| **325** | TTN c.50714G>A | VUS |  |  |
|  | TTN c.89708C>G | VUS |  |  |
|  | TTN c.104800C>T | P |  |  |
|  | TTN c.107033dupT | P |  |  |
| **326** | TTN c.50714G>A | VUS |  |  |
|  | TTN c.72766A>G | VUS |  |  |
|  | TTN c.98716G>A | VUS |  |  |
| **327** | TTN c.50801T>C | VUS | CLCN1 c.478C>T | P |
| **328** | TTN c.51055C>T | VUS | POLG c.1550G>T | VUS |
|  | TTN c.73825G>C | VUS |  |  |
| **329** | TTN c.51166C>T | LP |  |  |
| **330** | TTN c.51377A>G | VUS |  |  |
|  | TTN c.89471T>G | VUS |  |  |
| **331** | TTN c.51569G>T | VUS |  |  |
| **332** | TTN c.51712C>T | VUS |  |  |
|  | TTN c.3409G>C | VUS |  |  |
| **333** | TTN c.51809G>T | VUS | RETREG1 c.607G>A | VUS |
| **334** | TTN c.52381 C>T | VUS | ALS2 c.1641G>A | VUS |
|  |  |  | ATXN2 c.3077G>A | VUS |
| **335** | TTN c.52702A>G | VUS | COL6A2 c.2855C>T | VUS |
|  |  |  | COL6A3 c.5307G>C | VUS |
| **336** | TTN c.52702A>G | VUS | COL6A2 c.2950_2958dupGTGCTCACC | VUS |
|  | TTN c.86983G>A | VUS | POLG c.1760C>T | LP |
|  |  |  | POLG c.752C>T | LP |
|  |  |  | PYGM c.1537A>G | VUS |
| **337** | TTN c.52880G>A | VUS | COL6A3 c.7447A>G | P |
| **338** | TTN c.52880G>A | VUS |  |  |
|  | DHTKD1 c.1309G>T | P |  |  |
|  | LITAF c.44C>T | VUS |  |  |
|  | SH3TC2 c.581A>G | VUS |  |  |
| **339** | TTN c.53096G>A | VUS |  |  |
|  | TTN c.86104A>C | VUS |  |  |
| **340** | TTN c.53096G>A | VUS | PMP22 c.353C>T | LP |
|  | TTN c.79856G>A | VUS | PRX c.2548C>G | VUS |
|  |  |  | ZFYVE26 c.4789G>A | VUS |
| **341** | TTN c.53096G>A | VUS | POLG c.2243G>C | P |
|  | TTN c.80983G>A | VUS | SBF2 c.2323G>A | VUS |
|  |  |  | SH3TC2 c.3760A>G | VUS |
| **342** | TTN c.53096G>A | VUS | SH3TC2 c.505T>C | VUS |
| **343** | TTN c.53096G>A | VUS | NDRG1 c.1042G>A | VUS |
|  | TTN c.80983G>A | VUS |  |  |
| **344** | TTN c.53159T>C | VUS | SBF1 c.595C>T | VUS |
| **345** | TTN c.53660G>A | VUS | ATM c.2608A>G | VUS |
|  | TTN c.58333G>A | VUS |  |  |
|  | TTN c.59081G>A | VUS |  |  |
| **346** | TTN c.53843C>A | VUS | GARS1 c.929G>A | LP |
|  | TTN c.69130C>T | VUS |  |  |
| **347** | TTN c.54068G>A | VUS |  |  |
| **348** | TTN c.54160G>C | VUS | ZFYVE26 c.6T>A | VUS |
| **349** | TTN c.54710T>C | VUS | EGR2 c.192G>C | VUS |
| **350** | TTN c.54710T>C | VUS |  |  |
|  | TTN c.82691C>T | VUS |  |  |
| **351** | TTN c.54740T>C | VUS | ATXN7 c.916A>T | VUS |
|  | TTN c.66051G>A | VUS | CACNA1A c.1364G>A | LP |
|  |  |  | CREBBP c.2811G>A | VUS |
|  |  |  | POLG c.2028G>A | VUS |
|  |  |  | SBF2 c.2323G>A | VUS |
| **352** | TTN c.54796G>T | VUS |  |  |
| **353** | TTN c.55139T>C | VUS | COL6A3 c.5752C>T | VUS |
| **354** | TTN c.55374C>G | VUS | CREBBP c.6347A>G | VUS |
| **355** | TTN c.55374C>G | VUS | CACNA1A c.6238G>A | VUS |
|  | TTN c.69716-10dup | VUS | SH3TC2 c.505T>C | VUS |
| **356** | TTN c.55396G>A | VUS | ATM c.1229T>C | VUS |
|  | TTN c.43G>A | VUS | ATM c.5258A>G | VUS |
|  |  |  | DHTKD1 c.1079T>C | VUS |
|  |  |  | EPHB4 c.118G>A | VUS |
| **357** | TTN c.55619T>C | VUS |  |  |
|  | TTN c.70579G>A | VUS |  |  |
| **358** | TTN c.55627C>G | VUS |  |  |
| **359** | TTN c.55655G>A | VUS |  |  |
|  | TTN c.96904+4T>C | VUS |  |  |
| **360** | TTN c.55925T>A | VUS | ATM c.4324T>C | VUS |
|  | TTN c.77716G>A | VUS | ATM c.5005+7_5005+8del | VUS |
|  | TTN c.101665G>A | VUS | COL6A3 c.6520G>A | VUS |
| **361** | TTN c.56528C>G | VUS |  |  |
|  | TTN c.72166C>T | VUS |  |  |
| **362** | TTN c.56535G>A | VUS | PYGM c.1727G>A | P |
|  | TTN c.104251G>C | VUS |  |  |
| **363** | TTN c.56911G>A | VUS |  |  |
| **364** | TTN c.57400C>T | VUS | COL6A3 c.2972C>T | VUS |
|  |  |  | ZFYVE26 c.7417-5G>C | VUS |
| **365** | TTN c.57442A>G | VUS | PYGM c.2056G>A | P |
|  | TTN c.82691C>T | VUS |  |  |
| **366** | TTN c.57442A>G | VUS |  |  |
| **367** | TTN c.57559G>A | VUS | ATM c.7390T>C | VUS |
| **368** | TTN c.57655T>C | VUS |  |  |
|  | TTN c.106920G>A | VUS |  |  |
| **369** | TTN c.57860G>A | VUS |  |  |
| **370** | TTN c.58141G>A | VUS | ATM c.1229T>C | VUS |
| **371** | TTN c.58529G>A | VUS | CACNA1A c.6128C>T | VUS |
| **372** | TTN c.59319G>A | VUS |  |  |
|  | TTN c.106996C>G | VUS |  |  |
| **373** | TTN c.60007C>T | VUS | POLG c.2481-7C>T | VUS |
|  | TTN c.69130C>T | VUS |  |  |
| **374** | TTN c.60314T>G | VUS |  |  |
|  | TTN c.105562A>C | VUS |  |  |
| **375** | TTN c.60364G>A | VUS |  |  |
|  | TTN c.6420T>A | VUS |  |  |
| **376** | TTN c.60407T>A | VUS | COL6A3 c.4541T>C | VUS |
|  | TTN c.21712G>A | VUS |  |  |
| **377** | TTN c.60681T>A | VUS | PYGM c.148C>T | P |
|  |  |  | SBF2 c.3935G>A | VUS |
| **378** | TTN c.60721G>C | VUS | CHRNE c.506A>T | VUS |
|  | TTN c.61922G>A | VUS | PYGM c.160T>G | LP |
|  | TTN c.98294C>G | VUS |  |  |
| **379** | TTN c.60800A>G | VUS | CLCN1 c.501C>G | VUS |
|  |  |  | DHTKD1 c.467dup | P |
| **380** | TTN c.61051G>T | VUS | POLG c.2481-7C>T | VUS |
| **381** | TTN c.61409T>C | VUS |  |  |
| **382** | TTN c.61667G>A | VUS |  |  |
|  | TTN c.102275G>A | VUS |  |  |
| **383** | TTN c.61922G>A | VUS | PMP22 c.103G>A | VUS |
|  | TTN c.69130C>T | VUS |  |  |
|  | TTN c.98294C>G | VUS |  |  |
| **384** | TTN c.61922G>A | VUS |  |  |
|  | TTN c.69458A>C | VUS |  |  |
|  | TTN c.98294C>G | VUS |  |  |
| **385** | TTN c.61922G>A | VUS | CACNA1A c.6234C>A | VUS |
|  | TTN c.98294C>G | VUS | COL6A3 c.4156G>A | VUS |
|  |  |  | FXN c.280A>G | VUS |
|  |  |  | SH3TC2 c.3269C>G | VUS |
| **386** | **TTN c.61922G>A** | VUS |  |  |
|  | **TTN c.98294C>G** | VUS |  |  |
| **387** | TTN c.62097A>G | VUS | ZFYVE26 c.1415A>G | VUS |
|  |  |  | LITAF c.44C>T | VUS |
|  |  |  | NDRG1 c.*19G>A | VUS |
| **388** | **TTN c.62681G>T** | **VUS** | **ATM c.3630G>A** | **VUS** |
|  | **TTN c.66002C>G** | **VUS** |  |  |
| **389** | TTN c.62897G> | VUS |  |  |
| **390** | TTN c.63329C>T | VUS | ZFYVE26 c.4937G>A | VUS |
|  |  |  | ZFYVE26 c.7417-5G>C | VUS |
| **391** | TTN c.63439G>A | VUS | ZFYVE26 c.1184G>T | VUS |
|  |  |  | MPZ c.637G>C | VUS |
| **392** | TTN c.63439G>A | VUS | PRX c.185-1G>C | LP |
|  | TTN c.107576T>C | VUS |  |  |
| **393** | TTN c.63439G>A | VUS | ATM c.7375C>T | VUS |
|  | TTN c.107576T>C | VUS | CLCN1 c.899G>A | VUS |
| **394** | TTN c.63577C>T | VUS |  |  |
|  | TTN c.100400T>G | VUS |  |  |
| **395** | TTN c.63577C>T | VUS |  |  |
|  | TTN c.71036G>A | VUS |  |  |
|  | TTN c.97538G>A | VUS |  |  |
| **396** | TTN c.63589A>G | VUS | KMT2C c.6339A>C | VUS |
|  | TTN c.74600_74602delCAA | VUS | SH3TC2 c.31C>T | VUS |
|  | TTN c.94553T>C | VUS |  |  |
| **397** | TTN c.63589A>G | VUS | COL6A2 c.759A>G | VUS |
|  |  |  | OPA1 c.2218A>G | VUS |
|  |  |  | PRX c.1216G>A | VUS |
| **398** | TTN c.63868C>T | VUS | CHRNE c.593C>T | VUS |
|  |  |  | COL6A2 c.2580G>A | VUS |
|  |  |  | COL6A2 c.511G>A | VUS |
|  |  |  | EPHB4 c.1429G>A | VUS |
|  |  |  | ZFYVE26 c.3722G>A | VUS |
| **399** | TTN c.64051C>T | VUS | COL6A3 c.5387C>T | VUS |
|  |  |  | MORC2 c.286C>G | VUS |
|  |  |  | PYGM c.660G>A | VUS |
| **400** | TTN c.64898G>A | VUS | PYGM c.160T>G | VUS |
|  |  |  | SBF1 c.2672C>A | VUS |
| **401** | TTN c.65566G>A | VUS | ALS2 c.979A>G | VUS |
|  |  |  | CAV3 c.233C>T | VUS |
|  |  |  | MPZ c.637G>C | VUS |
| **402** | TTN c.66179A>G | VUS | SH3TC2 c.3686A>T | VUS |
| **403** | TTN c.66463+2dupT | LP | DHTKD1 c. 1364G>A | VUS |
|  | TTN c.99138G>C | VUS |  |  |
| **404** | TTN c.66601G>A | VUS |  |  |
| **405** | TTN c.66601G>A | VUS | KMT2C c.1355A>G | VUS |
|  | TTN c.1141G>A | VUS | PYGM c.645G>A | VUS |
| **406** | **TTN c.67099T>C** | VUS |  |  |
| **407** | TTN c.67099T>C | VUS |  |  |
| **408** | TTN c.67726G>A | VUS | COL6A2 c.1336G>A | VUS |
|  |  |  | DHTKD1 c.403T>G | VUS |
| **409** | TTN c.67975G>A | VUS |  |  |
| **410** | TTN c.68081G>A | VUS | COL6A2 c.1984G>A | VUS |
|  |  |  | KCNC3 c.1760C>G | VUS |
|  |  |  | PYGM c.-20C>T | VUS |
|  |  |  | ZFYVE26 c.1184G>T | VUS |
| **411** | TTN c.68458G>C | VUS | DHTKD1 c.1079T>C | VUS |
| **412** | TTN c.68458G>C | VUS | COL6A3 c.5968C>T | VUS |
|  |  |  | SBF2 c.1930-6T>G | VUS |
| **413** | TTN c.68458G>C | VUS | COL6A3 c.489G>A | VUS |
| **414** | **TTN c.68458G>C** | **VUS** | **COL6A3 c.4156G>A** | **VUS** |
|  |  |  | **NDRG1 c.122A>G** | **VUS** |
| **415** | TTN c.68458G>C | VUS | ATM c.2932T>C | VUS |
|  |  |  | CAV3 c.-37G>A | VUS |
|  |  |  | CLCN1 c.2364+10G>A | VUS |
|  |  |  | COL6A2 c.2683A>C | VUS |
|  |  |  | CREBBP c.6090G>A | VUS |
| **416** | TTN c.68458G>C | VUS | ZFYVE26 c.4401C>T | VUS |
|  | TTN c.78892G>A | VUS | ATXN7 c.2536A>G | VUS |
|  |  |  | SBF1 c.2396+7_2396+18delCAGGGACTGGGG | VUS |
| **417** | TTN c.68458G>C | VUS | FGD4 c.857G>C | VUS |
|  | TTN c.87412C>A | VUS | MORC2 c.286C>G | VUS |
|  |  |  | PYGM c.1094C>T | P |
| **418** | TTN c.68786C>T | VUS | CAV3 c.-37G>A | VUS |
|  |  |  | COL6A2 c.2585G>A | VUS |
|  |  |  | CREBBP c.6712G>A | VUS |
|  |  |  | IQSEC2 c.684C>T | VUS |
| **419** | TTN c.68824+5G>C | LP | COL6A3 c.9128G>A | VUS |
|  |  |  | TTR c.-61G>A | VUS |
| **420** | TTN c.68824+5G>C | LP | COL6A3 c.9128G>A | VUS |
| **421** | TTN c.68864G>C | VUS | ATM c.8395_8404del | P |
| **422** | TTN c.68864G>C | VUS | CHRNE c.1480_*48delinsC | VUS |
| **423** | TTN c.68864G>C | VUS | COL6A3 c.5369C>T | VUS |
| **424** | TTN c.69130C>T | VUS | KMT2C c.9931C>T | VUS |
| **425** | TTN c.69130C>T | LP | PMP22 c.353C>T | LP |
|  |  |  | PMP22 Duplication | P |
| **426** | TTN c.69130C>T | VUS | GARS1 c.302G>A | VUS |
|  |  |  | ZFYVE26 c.7055C>T | VUS |
| **427** | TTN c.69130C>T | VUS |  |  |
|  | DHTKD1 c.2185G>A | P |  |  |
| **428** | TTN c.69130C>T | VUS | MED25 c.1518_1519del | LP |
| **429** | TTN c.69130C>T | VUS | CACNA1A c.*639A>G | VUS |
|  | TTN c.81899G>A | VUS | COL6A3 c.2246del | P |
|  |  |  | TRPV4 c.733G>A | VUS |
| **430** | TTN c.69130C>T | VUS |  |  |
| **431** | TTN c.69460A>G | VUS | COL6A3 c.5610C>A | VUS |
|  | TTN c.91601A>T | VUS |  |  |
|  | TTN c.3100G>A | VUS |  |  |
| **432** | TTN c.69520T>G | VUS | COL6A2 c.1348G>C | VUS |
|  |  |  | GDAP1 c.817C>G | P |
|  |  |  | MED25 c.1004C>T | VUS |
|  |  |  | POLG c.2481-7C>T | VUS |
| **433** | TTN c.69716-10dup | VUS | CACNA1A c.6238G>A | VUS |
|  | TTN c.55374C>G | VUS | SH3TC2 c.505T>C | VUS |
| **434** | TTN c.69821G>A | VUS | ALS2 c.3046C>G | VUS |
|  |  |  | PYGM c.660G>A | VUS |
|  |  |  | TRPV4 c.29C>T | VUS |
|  |  |  | ZFYVE26 c.3722G>A | VUS |
| **435** | TTN c.69821G>A | VUS | CLCN1 c.2681G>A | VUS |
| **436** | TTN c.69821G>A | VUS |  |  |
| **437** | TTN c.69821G>A | VUS | ALS2 c.3158G>A | LP |
|  | TTN c.73825G>C | VUS | COL6A3 c.9524T>C | VUS |
|  |  |  | KMT2C c.5171G>T | VUS |
|  |  |  | NAGLU c.1744G>T | LP |
|  |  |  | PRX c.1216G>A | VUS |
| **438** | TTN c.69864A>G | VUS | KMT2C c.10747C>T | VUS |
| **439** | TTN c.70765G>C | VUS | CACNA1A c.5843-9_5843-8del | VUS |
|  | TTN c.73825G>C | VUS | SBF1 c.3147-8G>C | VUS |
|  |  |  | SBF1 c.4768A>G | VUS |
| **440** | TTN c.71222C>T | VUS | COL6A3 c.9061G>C | VUS |
|  | TTN c.6304G>T | VUS |  |  |
| **441** | TTN c.72132T>C | VUS |  |  |
| **442** | TTN c.72132T>C | VUS | KMT2C c.10763C>T | VUS |
|  |  |  | KMT2C c.6339A>C | VUS |
| **443** | TTN c.72132T>C | VUS | PYGM c.1827+1G>C | P |
|  | TTN c.93005G>T | VUS |  |  |
| **444** | TTN c.72132T>C | VUS | COL6A2 c.1336G>A | VUS |
|  |  |  | POLG c.1550G>T | VUS |
|  |  |  | SATB2 c.1802C>T | VUS |
| **445** | TTN c.72132T>C | VUS | KMT2C c.6339A>C | VUS |
| **446** | TTN c.72132T>C | VUS |  |  |
| **447** | TTN c.72146T>C | VUS | COL6A2 c.2998A>G | VUS |
|  |  |  | SH3TC2 c.3686A>T | VUS |
| **448** | TTN c.72766A>G | VUS |  |  |
|  | TTN c.86949A>G | VUS |  |  |
|  | TTN c.98716G>A | VUS |  |  |
| **449** | **TTN c.72766A>G** | **VUS** | **ATXN7 c.1141C>T** | **P** |
|  | **TTN c.98716G>A** | **VUS** | **SH3TC2 c.1942C>T** | **VUS** |
| **450** | TTN c.72766A>G | VUS | ATM c.6503C>T | VUS |
|  | TTN c.98716G>A | VUS | SBF1 c.4019C>T | VUS |
| **451** | TTN c.72826A>T | VUS | CLCN1 c.664G>A | VUS |
| **452** | TTN c.72836A>T | VUS | COL6A3 c.5501-4C>G | VUS |
|  |  |  | DHTKD1 c.209C>G | VUS |
|  |  |  | PYGM c.1970-6C>T | VUS |
| **453** | TTN c.73604C>A | VUS |  |  |
| **454** | TTN c.73705G>C | VUS | CLCN1 c.899G>A | VUS |
|  |  |  | COL6A3 c.3118G>A | VUS |
|  |  |  | KMT2C c.2684G>A | VUS |
| **455** | TTN c.73705G>C | VUS | COL6A2 c.2795C>T | VUS |
|  |  |  | COL6A3 c.9524T>C | VUS |
| **456** | TTN c.73825G>C | VUS | SBF1 c. 4892C>T | VUS |
|  | TTN c.6958C>T | VUS |  |  |
| **457** | TTN c.73825G>C | VUS | ALS2 c.4119A>G | VUS |
|  |  |  | COL6A3 c.5635G>A | VUS |
|  |  |  | PYGM c.1353dup | P |
| **458** | TTN c.73825G>C | VUS |  |  |
| **459** | TTN c.73825G>C | VUS |  |  |
| **460** | TTN c.73825G>C | VUS | CREBBP c.2811G>A | VUS |
| **461** | TTN c.73825G>C | VUS | COL6A3 c.3142A>G | VUS |
| **462** | TTN c.73825G>C | VUS |  |  |
| **463** | TTN c.73825G>C | VUS | MED25 c.1004C>T | VUS |
|  |  |  | TTN c.10018A>G | VUS |
| **464** | TTN c.73825G>C | VUS | KMT2C c.13633G>C | VUS |
| **465** | TTN c.73825G>C | VUS | ALS2 c.1115C>G | VUS |
|  |  |  | ALS2 c.979A>G | VUS |
|  |  |  | COL6A2 c.2795C>T | VUS |
|  |  |  | SBF2 c.2323G>A | VUS |
| **466** | TTN c.73825G>C | VUS | MED25 c.247C>G | VUS |
| **467** | TTN c.73994C>T | VUS | ALS2 c.1919G>A | VUS |
|  |  |  | FGD4 c.2039C>T | VUS |
|  |  |  | ZFYVE26 c.677G>A | VUS |
| **468** | TTN c.73994C>T | VUS | CLCN1 c.2680C>T | P |
| **469** | TTN c.74288A>G | VUS | PYGM c.645G>A | VUS |
| **470** | TTN c.74331C>T | VUS | ZFYVE26 c.30T>G | VUS |
| **471** | TTN c.74600_74602del | VUS | PMP22 Deletion | P |
|  |  |  | SH3TC2 c.3550A>G | VUS |
| **472** | **TTN c.74600_74602del** | **VUS** | **CHRNE c.1077_1079dup** | **VUS** |
|  |  |  | **KMT2C c.12794C>T** | **VUS** |
| **473** | TTN c.74600_74602delCAA | VUS |  |  |
|  | TTN c.94553T>C | VUS |  |  |
| **474** | TTN c.74600_74602delCAA | VUS | PRX c.3802G>C | VUS |
|  | TTN c.83315A>T | VUS | PRX c.3947C>T | VUS |
|  |  |  | SBF1 c.3922C>T | VUS |
| **475** | TTN c.74626G>A | VUS | SH3TC2 c.1768G>A | VUS |
|  | TTN c.87496G>A | VUS |  |  |
|  | TTN c.102328C>T | VUS |  |  |
| **476** | TTN c.75734G>A | VUS | ALS2 c.3703-10delC | VUS |
|  | TTN c.96522G>C | VUS | SBF1 c.5630C>T | VUS |
| **477** | TTN c.76088G>A | VUS | ZFYVE26 c.3299T>A | VUS |
| **478** | TTN c.76556T>C | VUS | DHTKD1 c.209C>G | VUS |
|  |  |  | KMT2C c.194A>T | VUS |
|  |  |  | PRX c.1436C>G | VUS |
|  |  |  | SBF1 c.3488G>A | VUS |
|  |  |  | ZFYVE26 c.7417-5G>C | VUS |
| **479** | TTN c.76740G>A | VUS | CREBBP c.2728A>G | VUS |
|  | TTN c.99340T>C | VUS | FGD4 c.50C>G | VUS |
| **480** | TTN c.77216C>G | VUS |  |  |
| **481** | TTN c.77216C>G | VUS |  |  |
| **482** | TTN c.77216C>G | VUS |  |  |
| **483** | TTN c.77216C>G | VUS | SH3TC2 c.1402_1403delinsTT | VUS |
|  |  |  | ZFYVE26 c.1184G>T | VUS |
| **484** | TTN c.77716G>A | VUS |  |  |
| **485** | TTN c.77716G>A | VUS | SH3TC2 c.3550A>G | VUS |
|  | TTN c.101665G>A | VUS |  |  |
| **486** | TTN c.77716G>A | VUS | SH3TC2 c.3550A>G | VUS |
|  | TTN c.101665G>A | VUS |  |  |
| **487** | TTN c.78148T>C | VUS | CACNA1A c.2410T>C | VUS |
|  |  |  | COL6A2 c.1333-10 C>G | VUS |
|  |  |  | SBF1 c.3560A>G | VUS |
|  |  |  | SBF2 c.1168A>G | VUS |
| **488** | **TTN c.78215G>A** | VUS | **COL6A3 c.2621G>A** | VUS |
| **489** | TTN c.78215G>A | VUS | DHTKD1 c.1364G>A | VUS |
|  |  |  | KMT2C c.12167A>G | VUS |
| **490** | TTN c.78890C>T | VUS | COL6A3 c.1688A>G | VUS |
|  | TTN c.2581G>C | VUS |  |  |
| **491** | TTN c.78892G>A | VUS | DHTKD1 c.923G>A | VUS |
|  |  |  | OPA1 c.113_130del | LP |
| **492** | TTN c.78896T>A | VUS | MPZ c.-35dupC | VUS |
|  |  |  | SBF1 c.55+8C>T | VUS |
| **493** | TTN c.79226G>A | VUS | OPA1 c.113_130del | LP |
| **494** | TTN c.79226G>A | VUS | GDAP1 c.556A>G | VUS |
|  |  |  | PRX c.1216G>A | VUS |
|  |  |  | RETREG1 c.607G>A | VUS |
| **495** | **TTN c.79249 C>T** | **LP** |  |  |
| **496** | TTN c.79546G>A | VUS |  |  |
| **497** | TTN c.80233G>C | VUS | COL6A2 c.511G>A | VUS |
|  |  |  | KMT2C c.779T>G | VUS |
|  |  |  | MFN2 c.880C>G | VUS |
| **498** | TTN c.80414A>C | VUS | ALS2 c.1816-8C>T | VUS |
|  |  |  | GARS1 c.1922A>G | VUS |
|  |  |  | SBF1 c.2967+8G>A | VUS |
| **499** | TTN c.80666A>G | VUS | CACNA1A c.6604C>T | VUS |
|  |  |  | GARS1 c.787G>A | VUS |
|  |  |  | MORC2 c.2680A>G | VUS |
| **500** | **TTN c.81527G>T** | **VUS** |  |  |
| **501** | TTN c.81635T>C | VUS | COL6A3 c.5610C>A | VUS |
|  | TTN c.6584A>G | VUS |  |  |
| **502** | TTN c.81647G>A | VUS | ATM c.3925G>A | VUS |
|  | TTN c.102271C>T | VUS |  |  |
|  | TTN c.5479G>T | VUS |  |  |
| **503** | TTN c.81673G>A | VUS | ZFYVE26 c.832A>G | VUS |
|  |  |  | CACNA1A c.6783+9G>A | VUS |
|  |  |  | NAGLU c.1346G>A | VUS |
| **504** | TTN c.81899G>A | VUS |  |  |
| **505** | TTN c.82691C>T | VUS | ATXN2 c.*18C>T | VUS |
|  |  |  | CLCN1 c.2680C>T | P |
|  |  |  | COL6A3 c.4117G>A | VUS |
| **506** | TTN c.82691C>T | VUS | COL6A2 c.2251G>A | VUS |
|  |  |  | COL6A2 c.2764G>T | VUS |
| **507** | TTN c.82754C>A | VUS | ATM c.6067G>A | VUS |
|  |  |  | CLCN1 c.899G>A | VUS |
|  |  |  | SBF1 c.55G>C | VUS |
| **508** | TTN c.82754C>A | VUS | POLG c.2207A>G | VUS |
| **509** | TTN c.82754C>A | VUS | COL6A2 c.1130G>A | VUS |
|  | TTN c.105037C>T | VUS | CREBBP c.7041C>G | VUS |
|  |  |  | DHTKD1 c.1079T>C | VUS |
|  |  |  | SBF1 c.2967+9C>T | VUS |
|  |  |  | SBF2 c.*59A>G | VUS |
|  |  |  | SBF2 c.3574A>G | VUS |
|  |  |  | TRPV4 c.28G>C | VUS |
| **510** | TTN c.83063G>A | VUS | SBF1 c.4927C>A | VUS |
| **511** | TTN c.83105G>A | VUS | MORC2 c.1603C>T | VUS |
| **512** | TTN c.83417G>A | VUS | ATXN3 c.916_917insAGCAGCAGCAGCAGCAGC | VUS |
| **513** | TTN c.83912C>T | VUS | KMT2C c.6339A>C | VUS |
|  | TTN c.97760G>A | VUS | PRX c.4115G>T | VUS |
|  |  |  | SBF1 c.55G>C | VUS |
| **514** | TTN c.83998G>T | VUS |  |  |
| **515** | TTN c.84871C>T | VUS |  |  |
| **516** | TTN c.84871C>T | VUS | SBF1 c.4927C>A | VUS |
| **517** | TTN c.84871C>T | VUS |  |  |
| **518** | TTN c.85573G>A | VUS |  |  |
| **519** | TTN c.85723G>A | VUS | ZFYVE26 c.*42G>C | VUS |
|  |  |  | ATM c.8147T>C | P |
|  |  |  | SH3TC2 c.3686A>T | VUS |
| **520** | TTN c.85976A>C | VUS |  |  |
| **521** | TTN c.86002A>G | VUS | ALS2 c.339C>T | VUS |
| **522** | TTN c.86321T>C | VUS | ZFYVE26 c.4301A>C | VUS |
|  |  |  | CACNA1A c.3268G>T | VUS |
| **523** | TTN c.86420G>A | VUS | KMT2C c.2571C>T | LP |
| **524** | TTN c.86654G>A | VUS | COL6A2 c.2696C>T | VUS |
| **525** | TTN c.86683G>A | VUS | ZFYVE26 c.3950C>T | VUS |
|  | TTN c.98892C>T | VUS | MED25 c.403A>C | VUS |
|  |  |  | SBF1 c.-7C>T | VUS |
| **526** | TTN c.86732_86734del | VUS | FGD4 c.1622A>T | VUS |
|  | TTN c.92696T>C | VUS |  |  |
| **527** | TTN c.87412C>A | VUS | COL6A3 c.5387C>T | VUS |
| **528** | TTN c.87412C>A | VUS | COL6A3 c.9128G>A | VUS |
|  | TTN c.106468T>C | VUS | KMT2C c.12239T>C | VUS |
|  |  |  | PYGM c.1957C>G | VUS |
|  |  |  | SBF2 c.5058A>T | VUS |
| **529** | TTN c.87412C>A | VUS | PYGM c.2280G>C | VUS |
| **530** | TTN c.87428G>T | VUS |  |  |
|  | TTN c.95647G>A | VUS |  |  |
| **531** | TTN c.88394C>T | VUS |  |  |
| **532** | TTN c.88394C>T | VUS | ALS2 c.4119A>G | VUS |
| **533** | TTN c.88394C>T | VUS | DHTKD1 c.-16G>C | VUS |
|  | TTN c.93803A>C | VUS |  |  |
|  | TTN c.105209C>T | VUS |  |  |
| **534** | TTN c.88394C>T | VUS | COL6A3 c.6998C>A | VUS |
|  | TTN c.98726T>C | VUS |  |  |
| **535** | TTN c.88394C>T | VUS | CLCN1 c.1437_1450del | P |
|  |  |  | OPA1 c.2441-4dup | VUS |
| **536** | TTN c.88394C>T | VUS |  |  |
| **537** | TTN c.88459G>A | VUS |  |  |
| **538** | TTN c.89405C>A | VUS |  |  |
| **539** | TTN c.89520C>T | VUS |  |  |
| **540** | TTN c.89520C>T | VUS | CLCN1 c.899G>A | VUS |
|  |  |  | GDAP1 c.248G>C | VUS |
|  |  |  | SBF1 c.5471G>A | VUS |
| **541** | TTN c.89989T>A | VUS |  |  |
| **542** | TTN c.90734C>T | VUS | CACNA1A c.3043G>A | VUS |
| **543** | TTN c.91075T>C | VUS |  |  |
| **544** | TTN c.91343G>A | VUS | NAGLU c.380A>G | VUS |
| **545** | TTN c.91601A>T | VUS |  |  |
|  | TTN c.3100G>A | VUS |  |  |
| **546** | TTN c.91643C>T | VUS | KMT2C c.12755C>T | VUS |
| **547** | TTN c.92042C>A | VUS | ATM c.4049C>T | VUS |
| **548** | TTN c.92042C>A | VUS | CLCN1 c.2680C>T | P |
|  |  |  | DHTKD1 c.1019G>A | VUS |
| **549** | TTN c.92059G>A | VUS | SH3TC2 c.3686A>T | VUS |
| **550** | TTN c.92167C>T | VUS | DHTKD1 c.1079T>C | VUS |
| **551** | TTN c.92176C>T | VUS |  |  |
| **552** | TTN c.92176C>T | VUS | CLCN1 c.501C>G | LP |
| **553** | TTN c.92176C>T | VUS | COL6A3 c.2879C>G | VUS |
|  |  |  | COL6A3 c.5224C>T | VUS |
|  |  |  | SBF2 c.2323G>A | VUS |
| **554** | TTN c.92207T>C | VUS | TRIO c.*97C>T | VUS |
|  |  |  | TRIO c.7348C>T | VUS |
| **555** | TTN c.93005G>T | VUS |  |  |
| **556** | TTN c.93005G>T | VUS | COL6A3 c.5681C>T | VUS |
|  |  |  | PMP22 Duplication | P |
|  |  |  | PYGM c.645G>A | VUS |
|  |  |  | SBF1 c.2879C>G | VUS |
|  |  |  | SH3TC2 c.137A>C | VUS |
| **557** | TTN c.93214C>T | VUS | CLCN1 c.2959A>G | VUS |
|  | TTN c.102428T>C | VUS | CREBBP c.1612A>G | VUS |
| **558** | TTN c.93803A>C | VUS | PYGM c.645G>A | VUS |
| **559** | TTN c.93900C>T | VUS | EPHB4 c.691C>T | VUS |
| **560** | TTN c.93968C>T | VUS | KCNC3 c.2236G>A | VUS |
| **561** | TTN c.94507G>A | VUS |  |  |
| **562** | TTN c.94711G>A | VUS |  |  |
|  | TTN c.98666C>A | VUS |  |  |
| **563** | TTN c.96230G>A | VUS | COL6A3 c.9245C>G | VUS |
| **564** | TTN c.96424G>A | VUS | COL6A2 c.565G>A | VUS |
| **565** | TTN c.97386C>T | VUS | CACNA1A c.6552G>A | VUS |
|  |  |  | NAGLU c.1401A>G | VUS |
|  |  |  | SBF1 c.188C>T | VUS |
|  |  |  | SBF2 c.2323G>A | VUS |
| **566** | TTN c.97760G>A | VUS | SBF2 c.2323G>A | VUS |
| **567** | TTN c.97760G>A | VUS | COL6A3 c.8243C>T | VUS |
| **568** | TTN c.97760G>A | VUS | KMT2C c.13016A>C | VUS |
|  |  |  | KMT2C c.2016A>T | VUS |
|  |  |  | KMT2C c.5792C>T | VUS |
| **569** | TTN c.97760G>A | VUS | ATXN2 c.643G>A | VUS |
|  |  |  | COL6A3 c.5833G>C | VUS |
|  |  |  | SBF2 c.4522C>T | VUS |
| **570** | TTN c.97760G>A | VUS | KMT2C c.10636C>G | VUS |
|  |  |  | RETREG1 c.607G>A | VUS |
|  |  |  | TRPV4 c.1392C>T | VUS |
|  |  |  | ZFYVE26 c.6817C>T | VUS |
| **571** | TTN c.97760G>A | VUS |  |  |
| **572** | TTN c.97978T>G | VUS | NDRG1 c.122A>G | VUS |
|  | TTN c.98267C>T | VUS | SH3TC2 c.505T>C | VUS |
| **573** | TTN c.98294C>G | VUS | CHRNE c.1180G>T | VUS |
|  |  |  | MED25 c.1210G>A | VUS |
| **574** | TTN c.98390A>G | VUS | ATM c.5269A>G | VUS |
| **575** | TTN c.98390A>G | VUS |  |  |
| **576** | TTN c.98390A>G | VUS | ZFYVE26 c.2585C>G | VUS |
|  |  |  | POLG c.2028G>A | VUS |
| **577** | TTN c.98614A>C | VUS | ALS2 c.1115C>G | VUS |
|  |  |  | TTR c.209G>A | VUS |
| **578** | **TTN c.99901G>A** | **VUS** |  |  |
| **579** | TTN c.99922G>A | VUS | COL6A2 c.759A>G | VUS |
| **580** | TTN C.99931C>T | P | NDRG1 c.1062_1091del | VUS |
| **581** | TTN C.99931C>T | P | NDRG1 c. 1062_1091del | P |
|  |  |  | SH3TC2 c.3762G>A | VUS |
